# The *Candida albicans* toxin candidalysin mediates distinct epithelial inflammatory responses through p38 and EGFR-ERK pathways

**DOI:** 10.1126/scisignal.abj6915

**Published:** 2022-04-05

**Authors:** Spyridoula-Angeliki Nikou, Chunsheng Zhou, James S. Griffiths, Natalia K. Kotowicz, Bianca M. Coleman, Mary J. Green, David L. Moyes, Sarah L. Gaffen, Julian R. Naglik, Peter J. Parker

**Affiliations:** 1 Protein Phosphorylation Lab, The Francis Crick Institute; London, UK; 2 Centre for Host-Microbiome Interactions, Faculty of Dentistry, Oral & Craniofacial Sciences, King’s College London; London, UK; 3 Division of Rheumatology and Clinical Immunology, University of Pittsburgh; Pittsburgh, USA; 4 Experimental Histopathology Lab, The Francis Crick Institute; London, UK; 5 School of Cancer and Pharmaceutical Sciences, New Hunt’s House, King’s College London; London, UK

## Abstract

The fungal pathogen *Candida albicans* secretes the peptide toxin candidalysin, which damages epithelial cells and drives an innate inflammatory response mediated by the epidermal growth factor receptor (EGFR) and mitogen-activated protein kinase (MAPK) pathways and the transcription factor c-Fos. In cultured oral epithelial cells (OECs), candidalysin activated the p38 MAPK signaling pathway, which resulted in heat shock protein 27 (Hsp27) activation, IL-6 release, and EGFR phosphorylation without influencing the induction of c-Fos. p38 activation was not triggered by EGFR but by two non-redundant pathways involving MAPK kinases (MKKs) and the kinase Src, which differentially controlled p38 signaling outputs. Whereas MKKs mainly promoted p38-dependent release of IL-6, Src promoted p38-mediated phosphorylation of EGFR in a ligand-independent fashion. In parallel, candidalysin also activated the EGFR-ERK pathway in a manner that depended on EGFR ligands, resulting in c-Fos activation and release of the neutrophil-activating chemokines G-CSF and GM-CSF. In mice, p38 was important for the early clearance events of oral *C. albicans* infection, but c-Fos was not. These findings delineate how candidalysin activates the p38 and ERK MAPK pathways that differentially contribute to immune activation during *C. albicans* infection.

## Introduction

*Candida albicans* is one of the most widespread human fungal pathogens and can cause superficial mucosal infections, including oropharyngeal candidiasis (OPC). OPC is highly prevalent in infants, patients with HIV/AIDS, diabetes, and iatrogenic or autoimmune-induced dry mouth (1–3) and is associated with an increased risk of esophageal cancer (4). *C. albicans* adopts several distinct cellular morphologies, most notably single-celled yeast and filamentous hyphal forms. The yeast-to-hypha transition is the major virulence determinant for mucosal infections (5,6). Mucosal epithelial cells can discriminate between these morphotypes through a biphasic immune mitogen-activated protein kinase (MAPK) pathway response (7,8). In oral epithelial cells (OECs), *C. albicans* yeast cells transiently activate all three MAPK pathways [c-Jun N-terminal kinase (JNK), extracellular signal−regulated kinases 1 and 2 (ERK1/2), and p38] and the activation of the transcriptional factor c-Jun, but this is not sufficient to induce inflammatory responses (7,9). However, in response to *C. albicans* hyphae, there is a sustained activation of all three MAPK pathways that results in c-Fos transcription factor activation, the release of inflammatory mediators such as interleukin-6 (IL-6) and the neutrophil-attracting chemokines granulocyte colony-stimulating factor (G-CSF) and granulocyte-macrophage colony-stimulating factor (GM-CSF), as well as the induction of cell damage (7). In mice, which do not harbor *C. albicans* as a commensal microbe, the first encounter with this fungus by OECs leads to recruitment and activation of innate immune cells, including IL-17–expressing *γδ* T cells and TCRαβ^+^ lymphocytes (collectively, innate Type 17 cells), macrophages, and neutrophils that work in concert to clear the infection (7,10,19,11–18).

We previously reported that both cell damage and the activation of epithelial immune responses during *C. albicans* invasion are driven by candidalysin, a cytolytic toxin secreted by *C. albicans* hyphae (20). Candidalysin is encoded by the *Extent of Cell Elongation 1 (ECE1)* gene (20) and, when secreted, it accumulates in the invasion "pocket" created by invasion of *C. albicans* hyphae into epithelial cells. In its pore-forming capacity, candidalysin causes membrane destabilization, calcium influx, as well as the release of lactate dehydrogenase (LDH), alarmins, and antimicrobial peptides (AMPs) such as S100A9 and beta-defensin 3 (BD3) (20,21).

The p38 MAPKs comprise a family of serine-threonine protein kinases that are important in mediating intracellular signaling and cytokine production during environmental stress and infections (22,23). Previous studies in OECs show that *C. albicans* activates c-Fos partially through p38 signaling (7) and that c-Fos and MAPK phosphatase 1 (MKP1) activation and inflammatory cytokine release also requires candidalysin activity and is mediated indirectly by the epidermal growth factor receptor (EGFR) (24). Therefore, it has been presumed that candidalysin promotes c-Fos induction by triggering p38 activation downstream of EGFR. However, while activation of MAPK cascades is highly complex and interconnected (25,26), EGFR is generally considered to activate the ERK1/2 pathway and not p38, while p38 can signal upstream of EGFR (27,28).

Here, we delineate the MAPK-mediated downstream activation events induced specifically by candidalysin in cultured OECs. Candidalysin induced p38 activation, driving heat-shock protein (Hsp27) activation, IL-6 release, and EGFR phosphorylation. Notably, p38 was not triggered by EGFR but was stimulated by a circuit requiring MAPK kinases (MKKs) or the kinase Src acting independently. In parallel, candidalysin activated ERK1/2 downstream of EGFR, resulting in c-Fos activation. Both the p38 and EGFR-ERK1/2 pathways promoted GM-CSF release independently. Finally, we demonstrated a role for p38, but not c-Fos, in early clearance of oral *C. albicans* infection in a murine OPC model. Taken together, this study delineates how the activation of specific MAPK pathways by candidalysin contributes to the production of distinct inflammatory mediators by OECs and highlights the importance of p38 in driving early clearance during oral *C. albicans* infection.

## Results

### Candidalysin triggers p38 signaling independent of the EGFR-ERK1/2-c-Fos pathway

**Fig. 1:**
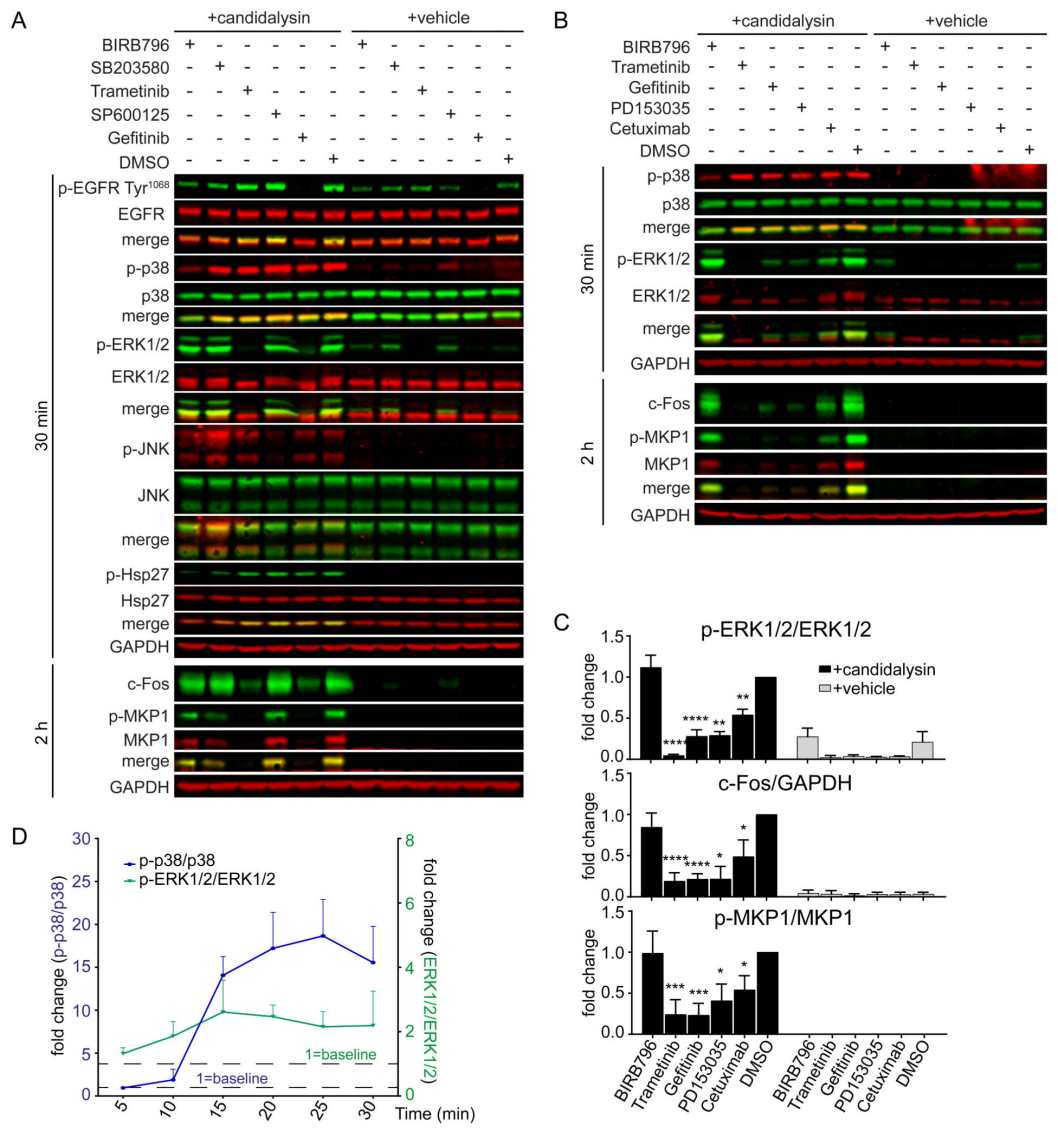
Candidalysin activates p38 independently of the EGFR-ERK1/2-c-Fos pathway. **A,** Representative immunoblot showing phosphorylated (p-) and total EGFR, p38, ERK1/2, JNK, and Hsp27 at 30 min and c-Fos induction and MKP1 phosphorylation at 2 h after candidalysin stimulation of TR146 OECs in the presence of BIRB796, SB203580, SP600125, Trametinib, Gefitinib, or DMSO. **B,** Representative immunoblot showing phosphorylated and total p38, ERK1/2, and MKP1 and c-Fos induction the indicated times after candidalysin stimulation in the presence of BIRB796, Trametinib, Gefitinib, PD153035, Cetuximab, or DMSO. Immunoblots are representative of three biological replicates (two for MKP1 in A). GAPDH is a loading control. **C,** Graphical quantification of immunoblots as in B. Graphs show means of three biological replicates + SD and are expressed as fold change relative to DMSO + candidalysin. **D,** Time-course of p38 and ERK1/2 phosphorylation following candidalysin stimulation. Data are means of three biological replicates + SD and are expressed as fold change relative to baseline amounts at 5 min post-candidalysin stimulation. Statistical significance for C was quantified by one sample t test compared to a hypothetical value = 1. *P < 0.05, **P < 0.01, ***P < 0.001, ****P < 0.0001.

To delineate host mechanisms of MAPK activation in response to candidalysin, we used TR146 cells, a tractable OEC model with high ultrastructural similarity to normal human buccal epithelium (29). We pretreated the cells with EGFR inhibitors (Gefitinib and PD153035), an EGFR ligand-binding neutralizing antibody (Cetuximab), a MEK1/2 inhibitor of the ERK1/2 pathway (Trametinib), a JNK inhibitor (SP600125), a p38α, β inhibitor (SB203580), or a p38α, β, γ, δ inhibitor (BIRB796) (22,30) prior to stimulating them with a lytic dose of candidalysin (70 μM), which has been shown to induce c-Fos activation and inflammatory cytokine release (20,24) (Fig. 1A-B). Candidalysin induced the phosphorylation of ERK1/2, JNK, EGFR, and p38, as well as phosphorylation of the downstream p38-dependent MAP kinase−activated protein kinase 2 (MK2) substrate Hsp27 (31–33) (Fig. 1A, fig. S1A), which occurred in a dose-dependent manner (fig. S1B-C).

Gefitinib and PD153035 strongly suppressed ERK1/2 and MKP1 phosphorylation as well as increases in c-Fos (Fig. 1B-C) but had no effect on p38 phosphorylation (Fig. 1A, fig. S1A), indicating that candidalysin does not activate p38 through EGFR and that EGFR appears to stimulate ERK1/2, MKP1, and c-Fos. Cetuximab significantly decreased ERK1/2 phosphorylation, MKP1 phosphorylation, and c-Fos induction (Fig. 1B-C) but to a lesser extent than did the EGFR tyrosine kinase inhibitors, suggesting that EGFR ligand binding to EGFR is an important but not an exclusive trigger of ERK1/2 activation in response to candidalysin. Moreover, TransAM assays, which quantify transcription factor binding to a consensus binding site in cell extracts, for c-Fos showed that Gefitinib and PD153035 strongly suppressed c-Fos DNA binding, whereas SB203580 had no effect (fig. S1D), consistent with the observed changes in c-Fos protein abundance (Fig. 1A-B). Accordingly, neither BIRB796 nor SP600125 influenced ERK1/2 phosphorylation (Fig. 1A), MKP1 phosphorylation, or c-Fos induction (Fig. 1A, fig. S1A), and BIRB796 had no effect on *FOS* mRNA expression (fig. S1E), confirming that c-Fos activation in OECs by candidalysin was promoted predominantly by the EGFR-ERK1/2 axis and not p38.

Confirming the observation that p38 activation was independent of the EGFR-ERK1/2 axis, Gefitinib treatment had no significant effect on Hsp27 phosphorylation (fig. S1A). We also monitored the time course of p38 and ERK1/2 activation in response to candidalysin (fig. S1F). Whereas ERK1/2 was phosphorylated immediately following candidalysin treatment and was maintained for the 30 min time course, p38 phosphorylation was delayed but rapidly increased from 15 min after stimulation (Fig. 1D). Collectively, these data indicate that p38 and ERK1/2 do not act in series and are activated asynchronously and independently by candidalysin.

### Candidalysin-induced p38 and EGFR-ERK1/2 activation trigger release of different cytokines

**Fig. 2:**
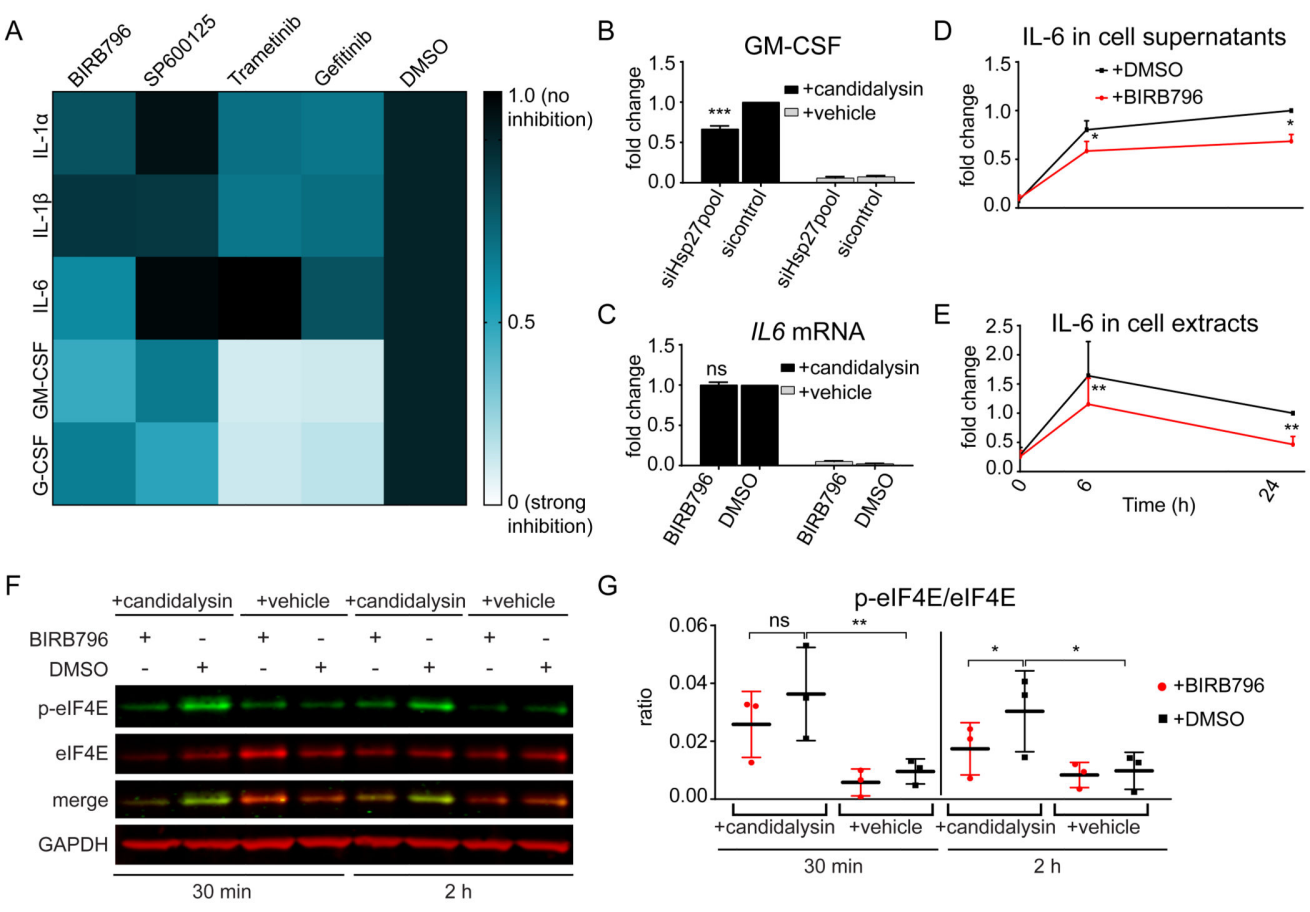
p38 and ERK1/2 trigger distinct patterns of cytokine release. **A,** Heatmap of the release of IL-1α, IL-1β, IL-6, GM-CSF, and G-CSF by TR146 OECs at 24 h post-candidalysin stimulation in the presence of BIRB796, SP600125, Trametinib, or Gefitinib. The heatmap was generated from the data in fig. S2A. **B,** Release of GM-CSF from TR146 cells transfected with a pool of Hsp27-targeted siRNAs 72 h prior to candidalysin stimulation for 24 h. Graph shows means of three biological replicates + SD and is expressed as fold change relative to siRNA control + candidalysin. **C,** Relative expression of *IL6* 6 h post-candidalysin stimulation in the presence of BIRB796. Graph shows means of three biological replicates + SD and is expressed as fold change relative to DMSO + candidalysin. **D and E,** IL-6 released into the supernatant D, and present in cell extracts E, before (0 h), after 6 h and after 24 h of candidalysin treatment with or without BIRB796. Graphs show means of three biological replicates + SD and are expressed as fold change relative to DMSO + candidalysin at 24 h. **F,** Representative immunoblot showing phosphorylated (p-) and total eIF4E 30 min and 2 h post-candidalysin stimulation in the presence of BIRB796. Immunoblots are representative of three biological replicates. GAPDH is a loading control. **G,** Graphical quantification of immunoblots as in F. Scatter plot shows the mean ± SD of three biological replicates expressed as ratios of p-eIF4E/eIF4E. Statistical significance for B and C was quantified by one sample t test compared to a hypothetical value = 1. Statistical significance in D, E and G was quantified by paired t-tests as indicated in the graphs. *P < 0.05, **P < 0.01, ***P < 0.001.

We next determined the contribution of MAPKs and EGFR to candidalysin-induced release of cytokines known to impact the progression of oral *C. albicans* infection in vivo (20,24). IL-1α exhibited a trend for reduction in the presence of Gefitinib and Trametinib, whereas IL-1β was significantly decreased, suggesting that these two cytokines are partially under the control of the EGFR-ERK1/2 axis (Fig. 2A, fig. S2A). Confirming previous results (24), Gefitinib and Trametinib completely blocked the release of GM-CSF and G-CSF, whereas BIRB796, SB203580, and SP600125 had more modest albeit significant inhibitory effects (Fig. 2A, fig. S2A). Although both p38 and ERK1/2 mediated GM-CSF release, this appeared to be implemented by different pathways: ERK1/2 through the activation of c-Fos (7) and p38 independently of ERK1/2 through a mechanism that may involve Hsp27 (Fig. 2B, fig. S2B-C). There is no direct evidence of a causative relationship between Hsp27 and GM-CSF release and although there is variation in the precise quantitative relationship between the two, there was a reduction in candidalysin-stimulated GM-CSF release following efficient knockdown for each siRNA/pool tested (Fig. 2B, fig. S2B-D). Finally, knockdown of individual p38 isoforms had no effect on cytokine induction (fig. S3A-C), indicating functional redundancy among p38 isoforms. These data indicate that EGFR-mediated activation of ERK1/2 signaling is the predominant MAPK pathway leading to release of GM-CSF and G-CSF in response to candidalysin.

Previously, p38α, β inhibition with SB203580 had been shown to have no effect on *C. albicans*−induced IL-6 release (7). However, in response to candidalysin, p38 was the only MAPK influencing IL-6 release, offering a potential biomarker of pathway function (Fig. 2A, fig. S2A). Notably, p38 (α, β, γ, δ) inhibition with BIRB796 had no effect on *IL6* mRNA expression, suggesting IL-6 production or release is controlled posttranscriptionally (Fig. 2C). Analysis of IL-6 protein in cells treated with BIRB796 showed a reduction in both supernatants and cell extracts even at 6 h after candidalysin stimulation, suggesting a translational rather than secretional input of p38 on IL-6 (Fig. 2D-E). To assess this, we investigated the involvement of p38 signaling in controlling the initiation of IL-6 translation through the MAPK-interacting protein kinases 1 and 2 (MNK1/2) and the eukaryotic initiation factor 4E (eIF4E) (34,35). BIRB796 reduced candidalysin-induced phosphorylation of eIF4E at 2 h post-stimulation (Fig. 2F-G), whereas it had no effect on MNK1/2 phosphorylation (fig. S2E-F), suggesting that p38 activation may control the translation of IL-6 through eIF4E but independently of MNK1/2.

### p38 contributes to candidalysin-induced EGFR phosphorylation

**Fig. 3:**
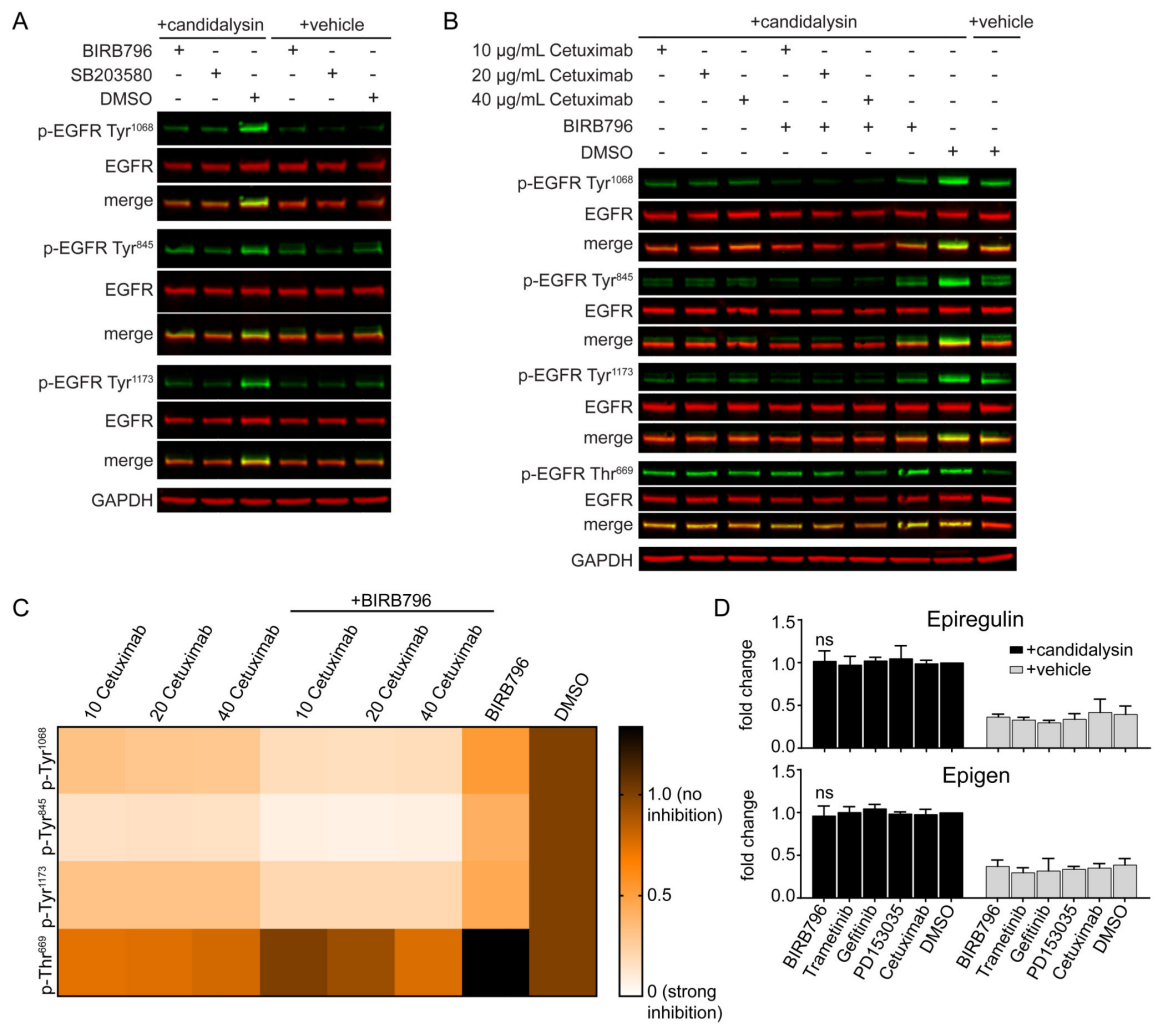
p38 contributes to candidalysin-induced EGFR phosphorylation in a ligand-independent manner. **A,** Representative immunoblot showing phosphorylation (p-) of EGFR on Tyr^1068^, Tyr^845^, and Tyr^1173^ in TR146 OECs 30 min post-candidalysin stimulation in the presence of BIRB796 or SB203580. **B,** Representative immunoblot showing phosphorylation of EGFR on Tyr^1068^, Tyr^845^, Tyr^1173^, and Thr^669^ 30 min post-candidalysin stimulation in the presence of various concentrations of Cetuximab, either alone or in combination with BIRB796. Immunoblots are representative of three biological replicates. GAPDH is a loading control. **C,** Heatmap showing EGFR phosphorylation on specific residues as in B. Heatmap was generated from quantitative data shown in the Supplementary Materials (fig. S4C). **D,** Release of epiregulin and epigen 30 min post-candidalysin stimulation in the presence of BIRB796, Trametinib, Gefitinib, PD153035, or Cetuximab, as measured by ELISA. Graphs show means of three biological replicates + SD and are expressed as fold change relative to DMSO + candidalysin. Statistical significance for D was quantified by one sample t test compared to a hypothetical value = 1.

Activation of p38 stimulates the phosphorylation of EGFR at Tyr^1068^, Tyr^845^ and Tyr^1173^, sites that are associated with EGFR receptor dimerization, autophosphorylation, and downstream MAPK activation (36–39). Candidalysin stimulated EGFR phosphorylation at these residues in a p38-dependent manner, and both p38 inhibitors, BIRB796 and SB203580, significantly reduced candidalysin-induced EGFR phosphorylation (Fig. 3A, fig. S4A-B). Most of the total phosphorylated p38 protein was p38α and p38β (fig. S3D-E); however, as noted above, isoform-specific siRNA knockdowns did not identify a specific p38 isoform that dominated EGFR phosphorylation, indicating redundancy (fig. S3A, C).

To delineate the individual roles of p38 versus EGFR ligands in EGFR activation, we assessed EGFR phosphorylation in TR146 cells pretreated with various concentrations of Cetuximab, an EGFR ligand-binding neutralizing antibody, alone or in combination with BIRB796, before candidalysin stimulation (Fig. 3B-C). Cetuximab treatment significantly, albeit incompletely, decreased EGFR phosphorylation at all three tyrosine residues. The use of high concentrations of Cetuximab (40 μg/mL) showed no additional effect over the lower doses (10 μg/mL, 20 μg/mL), suggesting that any remaining phosphorylation on these sites cannot be attributed to EGFR ligand-dependent mechanisms (fig. S4C). The use of BIRB796 alone also significantly reduced phosphorylation of all three tyrosine sites, but EGFR phosphorylation was near background amounts when the cells were pretreated with BIRB796 and Cetuximab in combination (fig. S4C). Because epiregulin and epigen are the only EGFR ligands released rapidly in high amounts after candidalysin stimulation (24) and because ERK1/2 is induced rapidly (Fig. 1D), it is possible that candidalysin-induced, p38-dependent phosphorylation of EGFR depends on p38 stimulating a rapid increase in ligand release. However, inhibition of p38 with BIRB796 had no effect on the release of these EGFR ligands (Fig. 3D).

EGFR phosphorylation on Thr^669^ is under the exclusive control of ERK1/2 (40,41), whereas Ser^1046/1047^ phosphorylation is triggered exclusively by p38 (42,43) (fig. S4D). Cetuximab treatment had no effect on Thr^669^ phosphorylation (Fig. 3B-C, fig. S4C), indicating that ligand binding to EGFR is not the only trigger for ERK1/2-dependent phosphorylation of EGFR. The ephrin type-A receptor 2 (EphA2) has been identified as a receptor for β-glucans on *C. albicans,* and knockdown of EphA2 is associated with suppressed EGFR phosphorylation and MEK1/2 activation (3,44). However, p38 inhibition with BIRB796 did not affect EphA2 phosphorylation, suggesting that p38-induced EGFR phosphorylation is not mediated by EphA2 activation (fig. S4E-F).

Collectively the data indicate that p38 does not signal downstream of EGFR but rather controls its phosphorylation through a ligand-independent mechanism that does not affect EGFR-mediated activation of the ERK1/2 pathway. The data also show that candidalysin-mediated activation of p38 induces distinct downstream markers, including IL-6 release, Hsp27, and EGFR-Tyr^1068^ phosphorylation but not c-Fos induction.

### MKK3 and MKK6 trigger candidalysin-induced p38 signaling

**Fig. 4:**
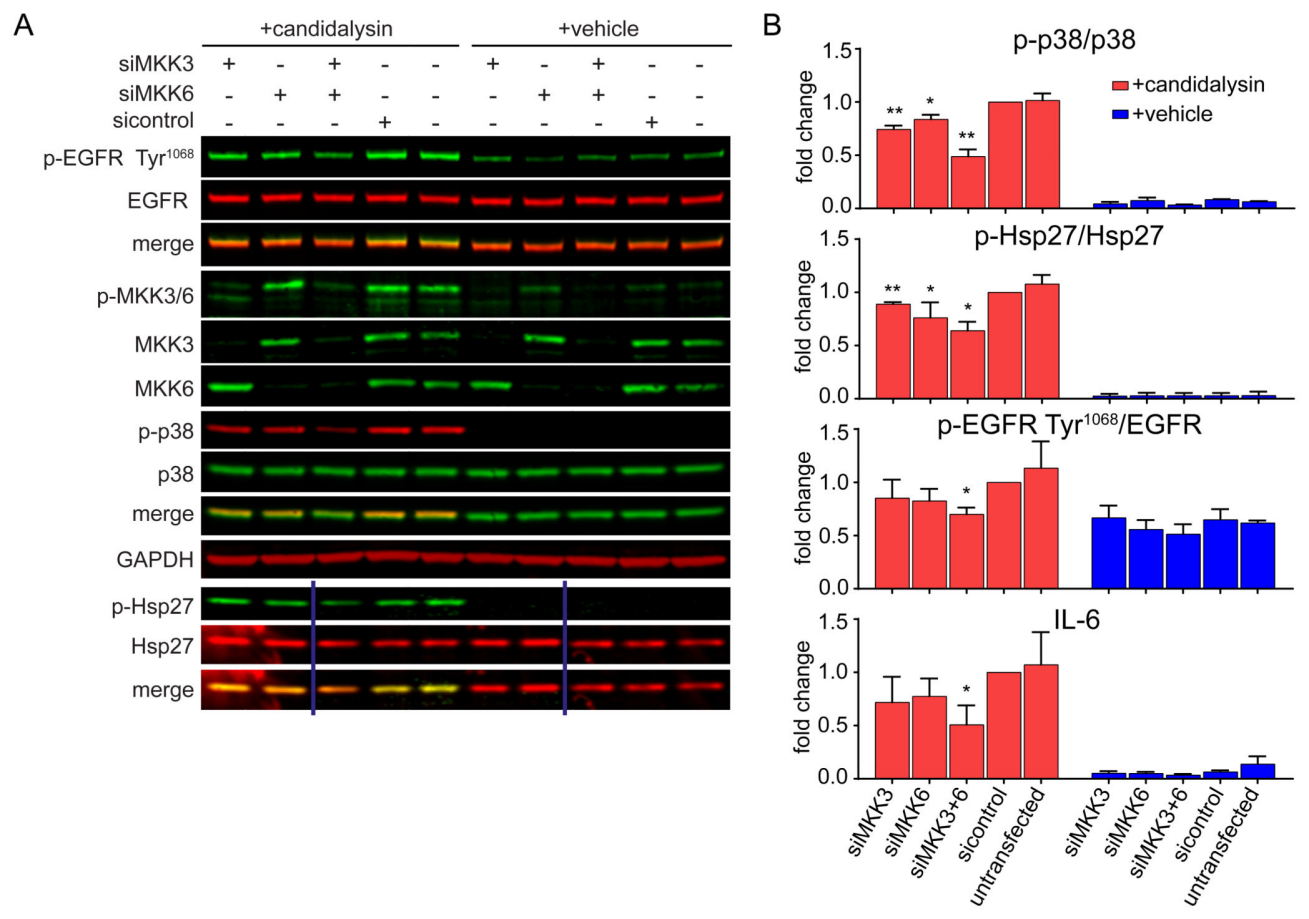
MKK3 and MKK6 trigger candidalysin-induced p38 signaling. **A,** Representative immunoblot showing phosphorylation (p-) of EGFR on Tyr^1068^, MKK3/6, p38, and Hsp27 and total MKK3 and MKK6 protein in TR146 OEC cells transfected for 72 h with siRNAs for MKK3 and/or MKK6 prior to candidalysin stimulation for 30 min. Immunoblots are representative of three biological replicates. GAPDH is a loading control. Vertical lines represent intervening gel lanes that are not shown. **B,** Graphical quantification of immunoblots as in A and IL-6 release from TR146 cells transfected for 72 h with siRNA for MKK3 and/or MKK6 prior to candidalysin stimulation for 24 h. Graphs show means of three biological replicates + SD and are expressed as fold change relative to siRNA control + candidalysin. Statistical significance was quantified by one sample t test compared to a hypothetical value = 1. *P < 0.05, **P < 0.01.

Given that candidalysin-induced p38 activation was independent of the EGFR-ERK1/2 axis, we next sought to delineate the mechanisms of p38 activation in response to the toxin. The canonical p38 pathway is activated by the upstream kinases MKK3 and MKK6 (22,45,46). Knockdown of MKK3 and MKK6 individually led to a significant decrease in candidalysin-induced p38 phosphorylation compared to siRNA control-treated cells, whereas combinatorial knockdown led to a greater reduction (Fig. 4A-B, fig. S5A), demonstrating a requirement for both MKKs for efficient p38 phosphorylation. To determine the effect of MKK3/6-mediated p38 activation, we measured the downstream effects of MKK knockdown. Hsp27 phosphorylation was partially reduced by either siRNA (Fig. 4A-B), whereas knockdown of both MKKs decreased EGFR phosphorylation at Tyr^1068^ and IL-6 release induced by the toxin (Fig. 4A-B), indicating the requirement of both MKK3 and MKK6 for the control of EGFR phosphorylation and IL-6 by p38.

### Src family kinases contribute to candidalysin-induced p38 activation independently of MKK3/6

**Fig. 5:**
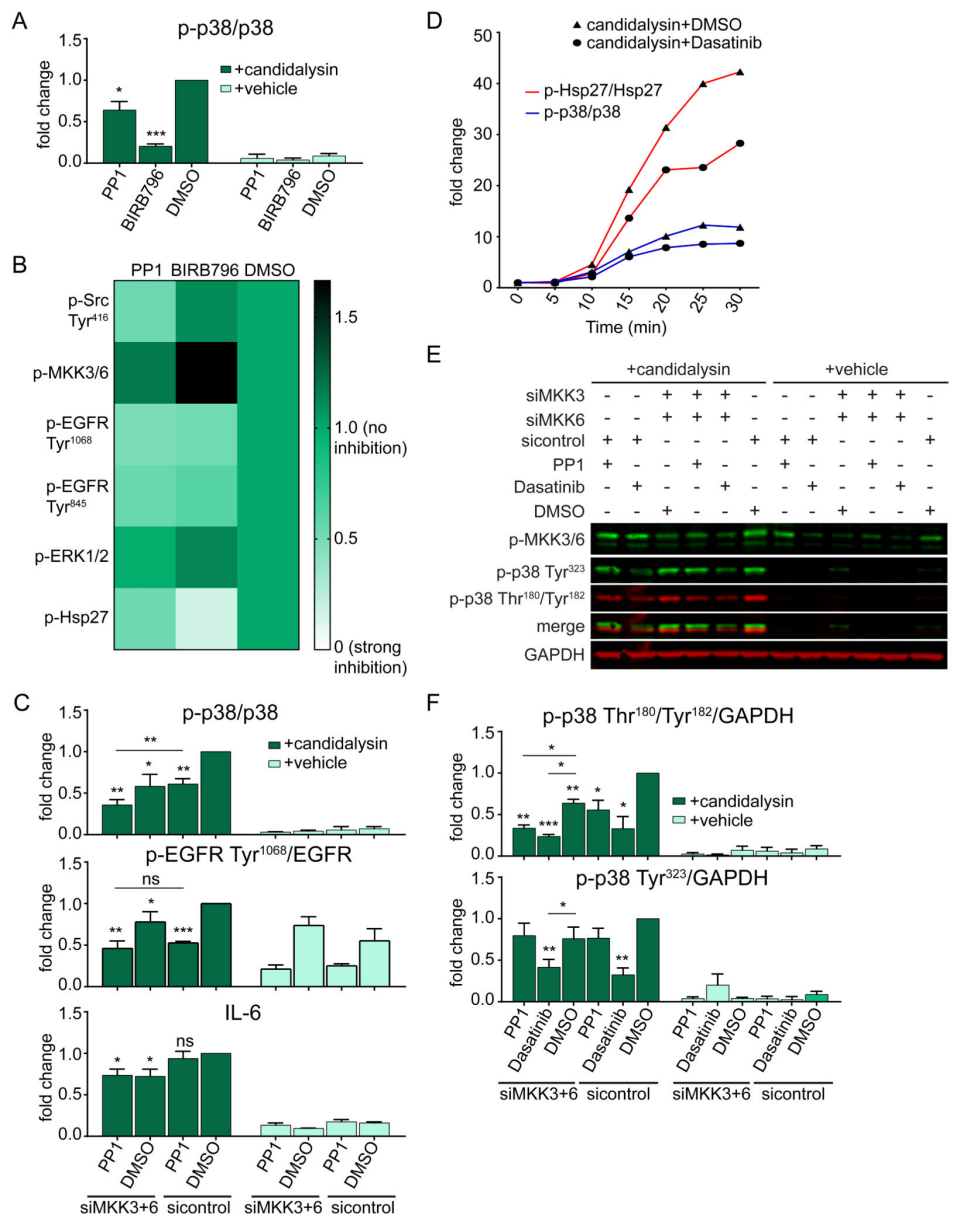
Src family kinases contribute to candidalysin-induced p38 activation with outputs distinct from MKKs. **A,** Graphical quantification of phosphorylated (p-) p38 in TR146 OEC cells treated with PP1 or BIRB796 prior to candidalysin stimulation for 30 min. Corresponding immunoblots are shown in the Supplementary Materials (fig. S6A). **B,** Visual representation of the amounts of phosphorylated Src (Tyr^416^), MKK3/6, EGFR (Tyr^1068^ and Tyr^845^), ERK1/2, and Hsp27 30 min post-candidalysin stimulation in the presence of PP1 or BIRB796. Corresponding immunoblots and quantitative data are shown in the Supplementary Materials (fig. S6, A and B). **C,** Quantification of phosphorylation of p38 and EGFR Tyr^1068^ and the release of IL-6 from TR146 cells transfected for 72 h with siRNA for MKK3 and MKK6 and/or treated with PP1 prior to candidalysin stimulation for 30 min or 24 h, respectively. Corresponding immunoblots are shown in the Supplementary Materials (fig. S6C). **D,** Graphical quantification of immunoblots (fig. S7C) showing phosphorylation of p38 and Hsp27 in TR146 cells treated with Dasatinib or DMSO and harvested at the indicated times post-candidalysin stimulation. Data are representative of three biological replicates and are expressed as fold change relative to baseline levels at 0 min post-candidalysin stimulation. **E,** Representative immunoblot showing phosphorylation of MKK3/6 and p38 on Tyr^323^ and Thr^180^/Tyr^182^ from TR146 cells transfected for 72 h with siRNA for MKK3 and MKK6 and/or treated with PP1 or Dasatinib prior to candidalysin stimulation for 30 min. Immunoblots are representative of three biological replicates. GAPDH is shown as a loading control. **F,** Quantification of immunoblots as in E. Graphs show means of three biological replicates + SD and are expressed as fold change relative to DMSO + candidalysin for A)or DMSO + siRNA control + candidalysin for C and F. Statistical significance in A was quantified by one sample t test compared to a hypothetical value = 1. Statistical significance in C and F was quantified by one sample t test compared to a hypothetical value = 1 and by paired repeated measures one-way ANOVA with Tukey’s multiple comparisons test as indicated in the graphs. *P < 0.05, **P < 0.01, ***P < 0.001.

Because MKK3/6 controlled only 50%-60% of p38 activation in response to candidalysin (Fig. 4B), we investigated whether MKK4 contributed to p38 activation in this context (45,47–49). Knockdown of MKK4 had no effect on candidalysin-induced p38 activation (fig. S5B-D), suggesting that MKK4 is likely not involved in candidalysin-stimulated p38 activation. We then focused on non-canonical p38 activation mechanisms and in particular Src family kinases (SFKs) (50–54). SFK inhibition using PP1, a broad-spectrum SFK inhibitor, significantly decreased p38 phosphorylation (Fig. 5A, fig. S6A). Notably, PP1 did not reduce phosphorylation of MKK3/6 (Fig. 5B, fig. S6B), suggesting that SFKs induce p38 phosphorylation through a parallel pathway independent of MKK3/6. In addition, phosphorylation of SFKs at a canonical site in the Src activation loop, Tyr^416^, (55) did not occur following candidalysin stimulation (fig. S6B). It is noted that cells maintained high basal phosphorylation of SFKs (fig. S6B). PP1 also reduced phosphorylation of EGFR and Hsp27 (Fig. 5B, fig. S6A-B), consistent with a role for SFKs in p38 activation. To investigate whether SFKs could transactivate EGFR and thereby activate ERK1/2 (56), we determined ERK1/2 phosphorylation in the presence of PP1. PP1 did not affect ERK1/2 phosphorylation in response to candidalysin (Fig. 5B, fig. S6B), confirming that p38 activation appears to function in a pathway parallel to EGFR signaling.

Although both MKK knockdown and PP1 pre-treatment reduced candidalysin-induced p38 phosphorylation, combinatorial treatment reduced p38 phosphorylation even further (Fig. 5C, fig. S6C). This suggests a non-redundant stimulation of p38 phosphorylation by MKK3/6 and Src (fig. S6D). Despite the additive effect of MKK3/6 and SFKs on p38 activation, SFKs were the dominant mediators in the p38-EGFR phosphorylation axis (Fig. 5C, fig. S6C), whereas MKK3/6 acted as regulators of IL-6 release through p38 (Fig. 5C). Hsp27 phosphorylation was controlled by both Src and MKK3/6 (fig. S6D). To identify the member of the Src family inducing p38 activation in this context, we used a structurally distinct and Src-selective inhibitor, Dasatinib (57), which yielded the same pattern of responses as did PP1 (fig. S7A-B), suggesting that Src is the member of the family that is likely to be recruited to mediate candidalysin-induced p38 activation through a MKK3/6 independent mechanism.

To further investigate the notion of MKK3/6 and Src differentially mediating p38 activation after candidalysin stimulation, we assessed the kinetics of p38 and Hsp27 phosphorylation induced by the toxin in the presence of Dasatinib (fig. S7C). Dasatinib reduced the phosphorylation of both p38 and Hsp27 starting at 5 min post-stimulation and continuing for the duration of the experiment, and the pattern of reduction was similar to the untreated cells and without fluctuations (Fig. 5D), suggesting that MKK3/6 and Src phosphorylate p38 throughout candidalysin stimulation and not sequentially. We next determined which p38 phosphorylation events were controlled by MKK3/6 versus Src (Fig. 5E-F). Although both MKK3/6 and Src induced p38 phosphorylation on Thr^180^-Tyr^182^, Src was the only mediator of p38 phosphorylation on Tyr^323^ (Fig. 5F), a phosphorylation event that leads to p38 autophosphorylation (58,59). Confirming these observations, immunoprecipitated p38α from lysates of candidalysin-stimulated cells displayed phospho-p38 Tyr^323^ (fig. S7D). Phosphorylation of p38α on Tyr^323^ was Src-dependent, because the amount of phospho-p38 Tyr^323^ associated with p38α was reduced when cells were pretreated with Dasatinib (fig. S7F). Reciprocally, immunoprecipitated phospho-p38 Tyr^323^ from lysates of candidalysin-stimulated cells revealed p38α (fig. S7E) which was reduced when cells were pretreated with Dasatinib (fig. S7F). Collectively, the data are supportive of a model in which candidalysin induces Src to phosphorylate p38 on Tyr^323^, thereby amplifying p38 signals.

Finally, the combination of BIRB796 (pan-p38 inhibitor) with PP1 or Dasatinib had no additive effect on the phosphorylation of EGFR, further supporting the role of Src in inducing EGFR phosphorylation through p38 (fig. S7G-H, Fig. 8). Collectively, these findings indicate that although both MKK3/6 and Src mediate candidalysin-induced p38 phosphorylation, these two independent pathways differentially control the signaling outputs of p38.

### p38 is required for early clearance of *C. albicans* during OPC independently of c-Fos

**Fig. 6:**
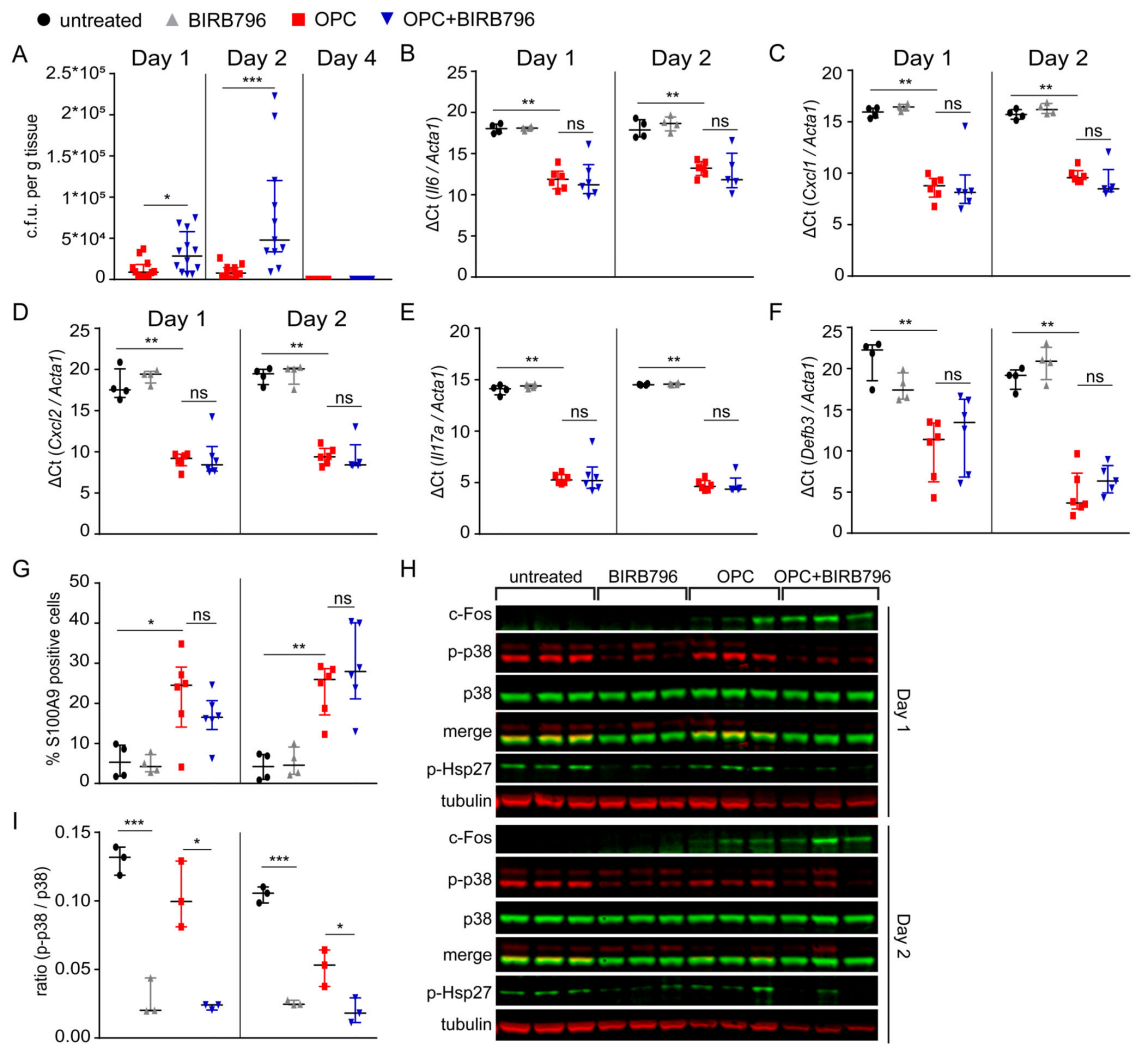
p38 is required for early clearance of *C. albicans* during OPC independent of c-Fos. Wild-type (Balb/c) immunocompetent mice were treated with either BIRB796 or vehicle control and orally inoculated with *C. albicans.* **A,** Fungal burdens from tongues of mice infected with *C. albicans* 1, 2 and 4 days post-infection (p.i.). Results are median ± interquartile range of 9-12 mice per group obtained over two independent experiments (one for day 4) (Mann-Whitney tests). **B to F,** Relative expression of *Il6* (B), *Cxcll* (C), *Cxcl2* (D), *Il17α* (E), and *Defb3* (F) in the mouse tongue homogenates 1 and 2 days p.i. mRNA amounts were determined by ΔCT method and normalized to *Acta1* (Mann-Whitney tests). **G,** Quantification of neutrophil recruitment (S100A9+ cells) in tongues of untreated, BIRB79- treated, and *C. albicans* infected mice with or without BIRB796 at 1 and 2 days p.i. Data is derived from densitometric analysis of immnohistochemical stains (fig. S8B). Results are median ± interquartile range of 4-6 mice per group obtained over two independent experiments (Mann-Whitney tests). **H,** Immunoblot showing c-Fos induction and p38 and Hsp27 phosphorylation in tongues from a single cohort of mice orally inoculated with *C. albicans* with or without BIRB796 and their respective controls at 1 and 2 days p.i. Tubulin is a loading control. **I,** Densitometric analysis of p38 phosphorylation as shown in (H). Results are median ± interquartile range of 3 mice per group from a single cohort of mice. (Welch’s unpaired t-tests). Each symbol represents one mouse. *P < 0.05, **P < 0.01, ***P < 0.001

Based on the differential activation of p38 by candidalysin and its independence from EGFR, we assessed its relevance in mounting the essential early innate Th17 response and immunity to OPC. To test this, immunocompetent wild-type (WT) mice were administered the pan-p38 inhibitor BIRB796 (p38α, β, γ, δ) (60) and weight loss and oral fungal burdens were assessed at days 1, 2 and 4 post-infection (p.i.). Treatment with BIRB796 increased fungal burden within tongue tissues at days 1 and 2 p.i. relative to controls (Fig. 6A), and the mice showed significant weight loss at day 1 p.i. (fig. S8A). However, by day 4, all mice fully cleared *C. albicans* and weight returned to normal, suggesting a role for p38 in early fungal clearance. Consistent with the candidalysin experiments in OECs (Fig. 2C), mice infected with *C. albicans* had increased *Il6* expression as indicated by the decreased ΔCt value and this was not affected by BIRB796 administration at either day 1 or 2 p.i. (Fig. 6B). Notably, treatment with BIRB796 did not affect the induction of *Cxcl1, Cxcl2,* or *Il17a* mRNA, or AMPs such as *Defb3* (encoding BD3) and S100a9 (confirmed by immunohistochemistry staining for S100A9^+^ cells) (Fig. 6C-G, fig. S8B). Moreover, the ratio of immature versus mature neutrophils in the bone marrow did not change, as shown by flow cytometry, confirming that fungal infection elicited a local rather than a systemic response (fig. S8C).

To confirm that BIRB796 impaired the p38 signaling pathway in local mucosal tissues, we monitored phosphorylation of the downstream p38 target, Hsp27 (Fig. 6H). Although untreated mice presented high baseline amounts of p38 phosphorylation, both p38 and Hsp7 phosphorylation were sensitive to BIRB796 (Fig. 6H-I). In contrast, *C. albicans-induced* c-Fos protein (Fig. 6H) and mRNA (fig. S8D) amounts of were not affected by p38 inhibition, confirming the independence of p38 signaling and c-Fos observed in OECs. In addition, induction of c-Fos at day 2 p.i. (Fig. 6H) did not correlate with increased ERK1/2 phosphorylation (fig. S8E-F), suggesting that additional proteins may be involved in c-Fos activation in vivo. Notably, the tongues tissues of infected mice did not produce IL-6 protein in detectable amounts (fig. S8E), suggesting that IL-6 may not be required for the acute responses against *C. albicans,* in agreement with a previous study (15). These data indicate that p38 signaling is independent of c-Fos during *C. albicans* infections, in agreement with the cell culture work.

**Fig. 7:**
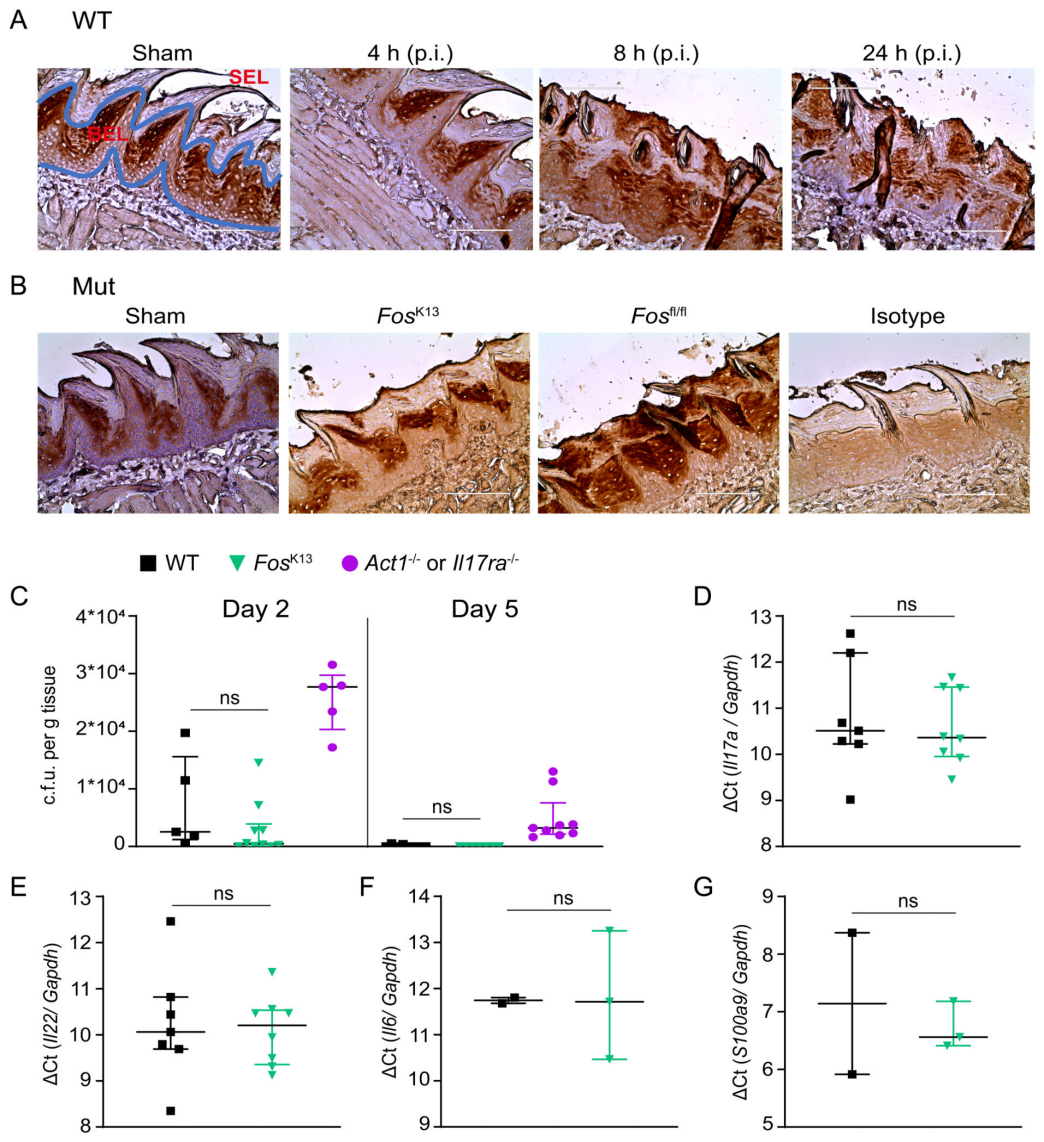
c-Fos induction is not required for host immunity against *C. albicans.* **A,** Wild-type mice (WT; C57BL/6) were subjected to sham infection (PBS) or OPC and tongues were harvested at the indicated time points post-infection (p.i.) for c-Fos staining by immunohistochemistry (IHC). SEL, suprabasal epithelial layer; BEL, basal epithelial layer. Scale bar, 100 μm. **B,** IHC for Fos in tongues of epithelial-specific *Fos* knockout (*Fos*^K13^) mice mock-infected (sham) or subjected to OPC. Tissues from infected unrecombined floxed control (*Fos*^fl/fl^) mice and tissues from infected Fos^K13^ mice stained with an isotype-matched antibody (isotype) are shown as positive and negative controls, respectively. Images are representative of at least two sections from individual mice. Scale bar, 100 μm. **C,** Fungal burdens from tongues of *Fos*^k13^ mice sublingually infected with *C. albicans* 2 and 5 days p.i. Results are median ± interquartile range of 5-10 mice per group obtained over two (for day 2) or three (for day 5) independent experiments (Mann-Whitney tests). **D to G,** Relative expression of *Il17a* (D), *Il22* (E), *Il6* (F), and *S100a9* (G) in mouse tongue homogenates 1 day p.i. mRNA amounts were determined by ΔCT method and normalized to *Gapdh.* Results are median ± interquartile range of 2-8 mice per group obtained over two for D and E or one for F and G independent experiments (Mann-Whitney tests). Each symbol represents one mouse.

Given that c-Fos is activated in the presence of candidalysin (20), and in order to assess its independence from p38 in vivo, we examined its role in a standard mouse OPC model (61). By immunohistochemistry c-Fos was produced tonically in the basal epithelial layer of the tongue (Fig. 7A). After infection with *C. albicans,* c-Fos was also observed in the suprabasal epithelial layer, starting at 8 h p.i. and sustained for at least 24 h (Fig. 7A). Suprabasal c-Fos was not seen after infection with the yeast-locked (non-virulent) *efg1*Δ/Δ strain (fig. S8G), suggesting that the yeast-hyphal transition is essential for c-Fos induction in the suprabasal layer. Thus, c-Fos is induced rapidly in the oral epithelium following infection with *C. albicans* in a hypha-dependent manner.

In order to elucidate the functional role of c-Fos in the superficial oral epithelium during OPC, we crossed *Fos*^fl/fl^ mice (62) to a strain expressing the Cre recombinase under the control of the murine keratin 13 (K13) promoter (hereafter termed *Fos*^K13^) (14). This system drives Cre expression in lingual, buccal, esophageal, and vaginal epithelial cells, without expression in skin or intestinal epithelia (14). We confirmed that c-Fos was not induced in the suprabasal epithelium in these mice, assessed at 24 h p.i. (Fig. 7B). *Fos*^K13^ mice were infected sublingually with *C. albicans* and weight loss and oral fungal burdens were assessed at days 2 and 5 p.i. (Fig. 7C, fig. S8H). Sham (PBS)-infected WT mice and *Il17ra^-/-^* mice were used as controls for infection clearance, respectively (63). No significant difference was observed in oral fungal burdens between WT and *Fos*^K13^ mice at day 2 p.i. (Fig. 7C), and both fully cleared *C. albicans* by day 5 showing no weight loss, whereas *Il17ra^-/-^* mice maintained oral fungal burdens and weight loss (Fig. 7C, fig. S8H). Moreover, infected *Fos*^K13^ mice showed normal induction of transcripts for *Il17a, Il22, Il6,* and AMPs such as *S100a9* at day 1 p.i (Fig. 7D-G). Thus, c-Fos–dependent induction of inflammatory cytokines was not required for *C. albicans* clearance.

Collectively, the data presented herein demonstrate that p38 signaling is independent of the EGFR-ERK1/2-c-Fos axis during murine *C. albicans* infections. The data also demonstrate a unique role for p38 in early and efficient fungal clearance but also indicate that c-Fos induction is not essential for host immunity against *C. albicans.*

## Discussion

**Fig. 8:**
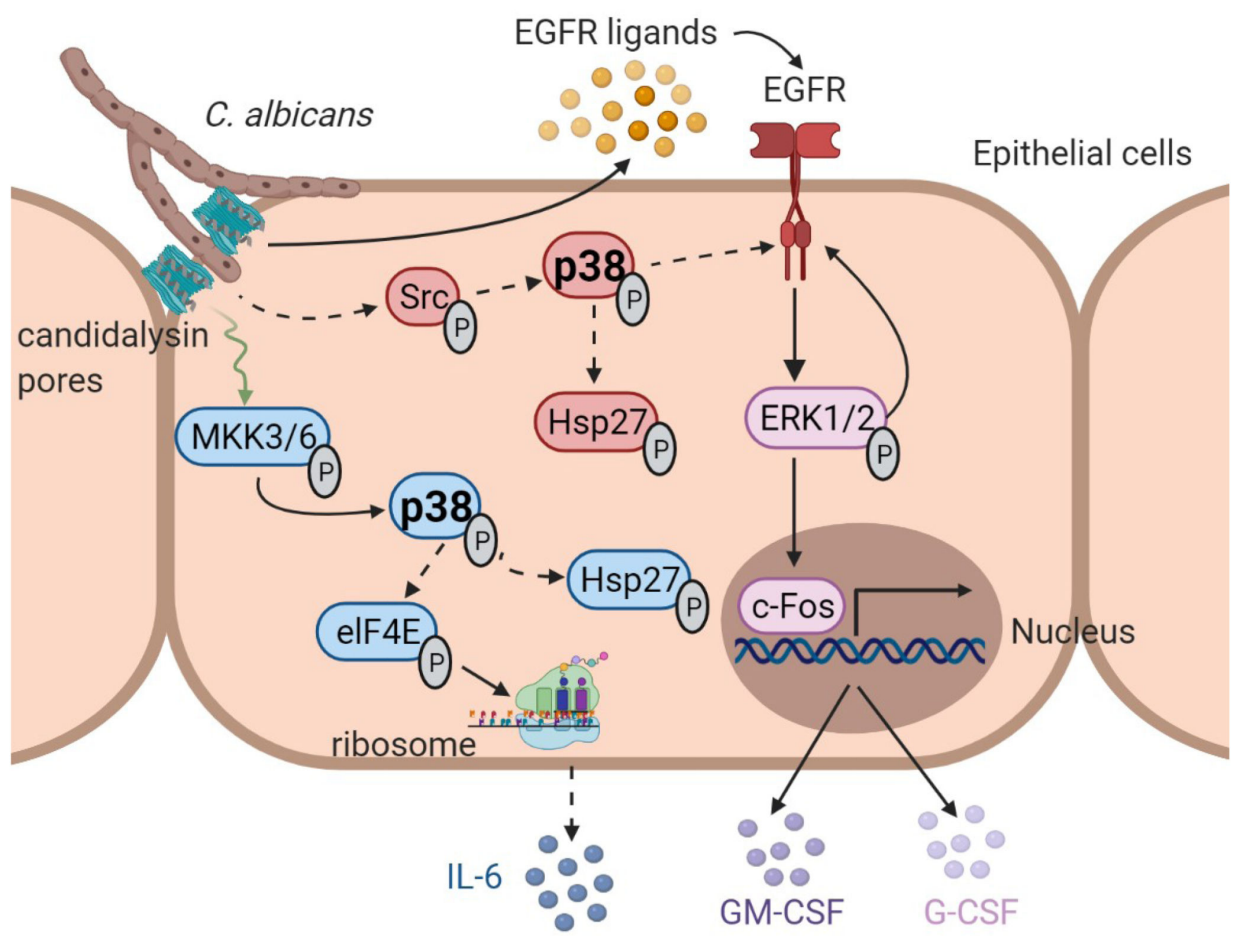
Graphical summary. Candidalysin is secreted into an invasion pocket formed by *C. albicans* hyphae. Candidalysin intercalates into the host cell membrane and stimulates the release of EGFR ligands, which induces ERK1/2 activation downstream of EGFR, resulting in c-Fos activation and the release of the neutrophil-activating chemokines GM-CSF and G-CSF. Through a parallel pathway, candidalysin also activates p38, resulting in IL-6 release and Hsp27 phosphorylation. p38 activation is not triggered by EGFR but by two selective and independent pathways that differentially control p38 signaling outputs. MKK3/6 promote IL-6 release, whereas Src triggers p38-induced EGFR phosphorylation independent of ligand binding. Both Src and MKK3/6 promote p38-dependent Hap27 phosphorylation. Created with BioRender.com

The discovery of a danger response pathway within OECs comprising MAPK signaling involving p38, ERK1/2, and c-Fos was critical to our understanding of how mucosal tissues respond to *C. albicans* hyphal invasion (7). The subsequent discovery that this host response was due to damage induced by the toxin candidalysin was a major advance in our understanding of fungal pathogenicity (20). These studies indicated that whereas *C. albicans* activated c-Fos partially through p38 signaling, c-Fos induction also required candidalysin activity and EGFR signaling (24). It was therefore presumed that c-Fos was activated by p38 downstream of EGFR, but how these signaling pathways were integrated to promote downstream effector responses was not defined. Here we aimed to delineate how candidalysin activates these MAPK signaling mechanisms. We found that candidalysin activated OECs by two distinct and independent pathways; EGFR-ERK1/2-c-Fos signaling and Src- or MKK3/6-p38 signaling, with both pathways mediating cytokine release (Fig. 8). The key discoveries are that candidalysin activates p38 to direct distinctive outputs independent of the EGFR-ERK1/2-c-Fos pathway, that p38 is triggered by two parallel pathways that independently determine its downstream effects, and that p38 signaling is protective during early stages of *C. albicans* infection, whereas c-Fos appears to be dispensable.

Candidalysin-induced p38 phosphorylation activated three downstream outputs: Hsp27 activation, IL-6 release, and EGFR phosphorylation. Hsp27 is an antiapoptotic protein located mainly in the cytoplasm, which under stress conditions interferes with apoptosomes, thereby inhibiting pro-Caspase-9 activity (32). Although transcriptomic and proteomic data have revealed the increased abundance of anti-apoptotic genes in response to *C. albicans* (64,65), Hsp27 phosphorylation was not previously known to occur during murine OPC infection. Our data suggest that Hsp27 might be involved in the release of GM-CSF, an anti-inflammatory cytokine that recruits phagocytic immune cells to clear infections (66–68). Although no direct link has been yet found between candidalysin-induced Hsp27 phosphorylation and GM-CSF release, studies implicate nuclear factor κB (NF-κB) in stimulating the transcription of *Csf2* (encodes GM-CSF) downstream of Hsp27 in macrophages (67,69).

Induction of epithelial signaling during *C. albicans* infection leads to release of cytokines that mediate host immunity (11,70,71). Here, we demonstrate that candidalysin induced IL-6 release in part through p38. Although IL-6 is dispensable for the development of innate Th17 cells during acute clearance of OPC (15), it is a useful biomarker for p38 pathway activity because p38 was the only MAPK that promoted its production following candidalysin stimulation. Although a previous study using SB203580 (p38α, β inhibitor) suggested that p38 is not involved in IL-6 release during *C. albicans* epithelial infection (7), using BIRB796 (p38α, β, γ, δ inhibitor) we showed that IL-6 was the only cytokine predominantly controlled by p38 in response to candidalysin. Notably, this differential response to *C. albicans* and synthetic candidalysin was also observed by Ho *et. al.,* where Gefitinib blocked IL-6 release during *C. albicans* infection but had no effect on release induced by candidalysin (24). Therefore, it appears that epithelial cells can discriminate between the synthetic and natural forms of candidalysin, likely as a result of how the toxin is presented to the cell, whether it contacts the entire epithelial surface or is secreted into an invasion pocket (72). Notably, the control of IL-6 by p38 may occur at the translational level though the stimulation of eIF4E phosphorylation, independent of MNK1/2. MNK1/2 can be phosphorylated by both p38 and ERK1/2 (73); however, ERK1/2 inhibition had no effect on IL-6 release, indicating that MNK1/2 are not involved in IL-6 release by candidalysin. Indeed, eIF4E can be phosphorylated by other pathways downstream of p38 independently of MNK1/2 (74–76). In addition, the lack of transcriptional regulation of *IL6by* p38 indicates that other pathways, such as NF-κB, may promote the induction of *IL6* expression (7) or posttranscriptional modifications (77) by candidalysin.

EGFR and ERK1/2 were the main pathways controlling GM-CSF and G-CSF secretion. Although p38 also contributed to GM-CSF and G-CSF release, this was independent of EGFR, because the EGFR inhibitor Gefitinib had no effect on p38 phosphorylation or its downstream targets Hsp27 and IL-6. Notably, however, p38 inhibition was able to suppress candidalysin-induced phosphorylation of EGFR at multiple tyrosine sites. Given that both p38 inhibitors had the same effect, this suggests that p38α and β are the dominant isoforms mediating EGFR phosphorylation. Tyr^1068^ and Tyr^1173^ are autophosphorylation sites, and attenuation of their phosphorylation by p38 inhibitors indicates that p38 may modulate EGFR dimer formation (39,78) or EGFR trafficking (78). The Ser^1046/1047^ site is associated with EGFR ubiquitylation and subsequent degradation (42,43). Although p38 inhibition attenuated phosphorylation at this site, candidalysin did not appear to induce its phosphorylation in the first place (fig. S4D), suggesting that candidalysin-induced EGFR phosphorylation does not lead to degradation of the receptor. Although the mechanism through which p38 mediates EGFR phosphorylation is unclear, it does not appear to involve the β-glucan receptor EphA2. Moreover, unlike ligand-dependent EGFR phosphorylation, p38-dependent EGFR phosphorylation did not trigger downstream signaling responses through ERK1/2. p38-dependent EGFR phosphorylation is associated with clathrin-mediated EGFR endocytosis (28,79–81) and the endocytosed receptor does not contribute to ERK1/2 signaling (82), suggesting therefore that p38 inhibition may retain EGFR on the plasma membrane where it can more effectively contribute to ERK1/2 signaling during candidalysin stimulation.

Based on the studies of Moyes *et al.* and Westman *et al.* (20,83), it is self-evident that candidalysin intercalates into the plasma membranes of host cells. However, no host receptor for candidalysin has yet been identified on epithelial cells. All pore-forming toxins induce p38 activation as a result of potassium efflux upon plasma membrane damage (84–86). Indeed, p38 is a sensitive detector of even extremely low doses of these toxins, and its activation can be inhibited by osmotic stress relief (87). Candidalysin-induced p38 activation is mediated by the upstream kinases MKK3/6 and Src, independently of EGFR. MKK3 and MKK6 contributed approximately 50% of the p38 phosphorylation, whereas no evidence for a contribution by MKK4 was observed. Given this, we also investigated non-canonical mechanisms of p38 phosphorylation, which include the transforming growth factor-β-activated protein kinase 1 (TAK1)-binding protein (TAB1) and SFKs (58,59,88–90). Among these, we focused on SFKs in p38 activation because Src can act as an upstream activator of MKK3/6 (51,53,54,91) and is associated with EGFR phosphorylation in response to *C. albicans* (92). The SFK member Lck induces phosphorylation of p38 at Tyr^323^ in the absence of upstream MKKs, which triggers autophosphorylation (58,59). We found that Src could activate p38 simultaneously with but independently of MKK3/6. Moreover, candidalysin did not trigger Src phosphorylation, suggesting that Src is perhaps recruited to enhance p38 phosphorylation. Notably, Src triggered p38-mediated EGFR phosphorylation on Tyr^1068^ more potently than did MKK3/6. In response to candidalysin, Src-activated p38 was the dominant route to p38-promoted phosphorylation of multiple sites on EGFR (Fig. 3C, 5B), including a site that has been shown previously to also be a direct target of Src (56,93,94). In parallel, MKK-activated p38 is the principal pathway targeting IL-6 release. This pattern of behavior indicates that there are spatially separated signaling pathways leading to distinct differential immune outputs (Fig. 8).

Two previous in vivo studies using the same murine OPC model employed here showed that EGFR inhibition leads to decreased fungal burden (24,95), with EGFR having dual functions: a disease-promoting role enhancing fungal endocytosis by the adhesin Als3 (95) and a disease-protecting role mediating immune responses after candidalysin activation (24). Because c-Fos is under the control of EGFR, the data raised the possibility that c-Fos may play a critical role during OPC. However, using *Fos*^K13^ conditional knockout mice, we demonstrated that c-Fos was dispensable for enhancing fungal clearance and protective responses. In contrast, p38 signaling protected during OPC but only during early stages of infection. Given that p38 also acts on EGFR independently of ERK1/2-c-Fos, this supports a role for the p38-EGFR (rather than the EGFR-ERK1/2-c-Fos) axis in the early protection during OPC as a result of candidalysin exposure. Combined, these studies indicate that candidalysin targets EGFR-ERK1/2-c-Fos signaling and p38 signaling independently, and that p38 activation by candidalysin may act as an important regulator of controlled fungal invasion and host homeostasis during acute oral *C. albicans* infection.

In summary, our study demonstrates that candidalysin activated p38 independently of EGFR, resulting in IL-6 release and Hsp27 phosphorylation. p38 was not triggered by EGFR but by two selective pathways, mediated by MKK3/6 and Src, in a non-redundant fashion. In parallel, candidalysin induced ERK1/2 activation through EGFR-ligand binding, resulting in c-Fos activation and the release of neutrophil-activating chemokines (Fig. 8). Finally, we identified p38 signaling as an important component of host homeostasis during the acute phase of mucosal *C. albicans* infections.

## Materials and Methods

### Chemical reagents and antibodies

Chemical reagents used in this study were BIRB796 (10 μM, #S1574, Selleckchem), SB203580 (10 μM, #S1076, Selleckchem), Gefitinib (2 μM) and Trametinib (30 nM) (kind gifts from the Oncogenic Biology Lab, the Francis Crick institute), SP600125 (20 μM, kind gift from the Cancer Epigenetics lab, the Francis Crick institute), Dasatinib (300 nM, #S1021, Selleckchem), PD153035 (500 nM, #S6546, Selleckchem), PP1 (10 μM, #0040, Merck), and Cetuximab (10 μg/mL, Merck KGaA, kind gift from the cancer centre of Guy’s Hospital). Primary antibodies used were purchased from Cell Signalling Technology unless otherwise stated and include p38 MAPK (#8690), p38 MAPK (#ab31828, Abcam), phospho-p38 MAPK Thr^180^/Tyr^182^ (#9216), p38α MAPK (#9218), p38β MAPK (#2339), p38γ MAPK (#2307), p38δ MAPK (#2308), EGFR (#E3138, Merck), phospho-EGFR Tyr^1068^ (#3777), Tyr^1173^ (#4407), Ser^1046/1047^ (#2238), Thr^669^ (#8808), Tyr^845^ (#2231), SAPK/JNK (#9252), phospho-SAPK/JNK Thr^183^/Tyr^185^ (#9255), p44/42 MAPK Erk1/2 (#9107), phospho-p44/42 MAPK Erk1/2 Thr^202^/Tyr^204^ (#4370), MAPKAPK-2 (#3042), c-Fos (#2250), MKP1 (#ab236501, Abcam), phospho-DUSP1/MKP1 Ser^359^ (#2857), MKK3 (#8535), MKK6 (#8550), SEK1/MKK4 (#9152), phospho-MKK3 Ser^189^/MKK6 Ser^207^ (#12280), non-phospho-Src Tyr^416^ (#2102), phospho-Src Tyr^416^ (#MAB2685-SP, R&D systems), Hsp27 (#2402), phospho-Hsp27 Ser^82^ (#9709), EphA2 (#12927), phospho-EphA2 Ser^897^ (#6347), phospho-eIF4E Ser^209^ (#9741), eIF4E (#MAB3228, R&D Systems), phospho-MNK1/2 Thr^197/202^ (#2111), IL-6 (#ab208113, Abcam), normal rabbit IgG (#2729), phospho-p38 Tyr^323^ (#PA5-105007, Thermo Fisher Scientific), GAPDH (#AB2302, Merck Millipore), tubulin (#T5168-100UL, Merck Millipore) and LI-COR IRDye 800CW/680RD secondary antibodies (#926-3221 / #926-68070, LI-COR).

### Cell culture and treatment with candidalysin

TR146 human buccal epithelial squamous cell carcinoma cells acquired from the European Collection of Authenticated Cell Cultures (ECACC) were cultured in Dulbecco’s modified Eagle medium/Nutrient Mixture F-12 (DMEM/F12) (Gibco, ThermoFisher Scientific) supplemented with 10% (w/v) fetal bovine serum (FBS; Gibco, ThermoFisher Scientific) and 1% penicillin-streptomycin (Gibco, ThermoFisher Scientific). The cells were maintained at 37°C in the presence of 5% CO_2_ and were regularly monitored for Mycoplasma infection. Prior to candidalysin treatment, confluent TR146 cells were serum-starved overnight, and all experiments were carried out in serum-free DMEM/F12. The candidalysin concentration used was 70 μM unless otherwise stated. As a negative control, TR146 cells were mock-"infected" with 1:40 deionized water (vehicle). For the experiments where inhibitors were used, these were added 1 h prior to candidalysin stimulation.

### Cell lysis and Western blotting

TR146 cells were lysed using a modified RIPA lysis buffer (50 mM Tris-HCl pH 7.4, 150 mM NaCl, 1 mM EDTA, 1% Triton X-100, 1% sodium deoxycholate, 0.1% SDS) containing protease (#05892791001, Merck) and phosphatase (#PHOSS-RO, Merck) inhibitors, left on ice for 30 min and then centrifuged for 10 min at 13000 rpm at 4°C. Cleared total protein lysates content was determined using the BCA protein assay kit (#23227, ThermoFisher Scientific). 15 μg of total protein was separated on 4-12% Bis-Tris gels (Invitrogen) before transfer to PVDF membranes (Merck Millipore) and blocking for 1 h with LI-COR blocking buffer. Membranes were then incubated in indicated primary antibodies in blocking buffer with 0.2% Tween 20 overnight at 4°C. Membranes were washed with TBS-T and probed in 1:10,000 IRDye 800CW and 680RD secondary antibodies with 0.2% Tween 20 for 1 h before being washed again and imaged using the LI-COR Bioscience Odyssey CLx Imager. Quantification of band density was conducted using the Image Studio Lite software. Human GAPDH was used as a loading control.

### Immunoprecipitation

TR146 cells were seeded in 10 cm dishes and adjusted to the density of 4 x 10^5^ cells/ml. Following experimentation, 1:100 PMSF was added in the modified RIPA lysis buffer, including protease and phosphatase inhibitors, and cell lysates were generated as described above. After the centrifugation step a small aliquot of lysate was taken to check the initial levels of the desired protein prior to immunoprecipitation (total lysate before IP). Protein G Dynabeads magnetic beads (#10003D, Thermo Fisher Scientific) were pre-washed in order to reduce non-specific protein binding to the beads and IgG. Subsequently, beads were resuspended in the original volume of 20 μl and added in the lysate along with 2 μg of IgG antibody. Lysates were then incubated on a rotating wheel at 4°C for 30 min and the supernatant was magnetically separated from the beads. p38α or phospho-p38 Tyr^323^ primary antibody was added 1:50 in the cleared supernatants and samples were incubated on a rotating wheel at 4°C overnight. The following day, 20 μl of protein G Dynabeads magnetic beads were added in the samples prior to incubation with rotation for 1.5 h at 4°C. Beads were magnetically separated and washed three times in lysis buffer and subsequently the immunoprecipitated protein was resuspended in 20 μl of lysis buffer (lysate after IP) and eluted through the addition of 20 μl sample buffer followed by boiling at 95°C for 5 min. Resulting samples were then separated by Western blotting.

### Cytokine determination

Following experimentation, cell culture supernatants were collected and centrifuged at 13000 rpm for 10 min. Cytokine levels in cleared supernatants were determined using the Magnetic Luminex® Performance Assay Human Base Kit A (#LUHM000, R&D systems) and a Bioplex 200 machine according to the manufacturer’s instructions. The data were analysed using Bioplex Manager 6.1 software to determine analyte concentrations.

### RNA Interference

Knockdown of gene expression was performed using ON-TARGETplus siRNAs for *MAPK14* (#L-003512-00-0005), *MAPK11* (#L-003972-00-0005), *MAPK12* (#L-003590-00-0005), *MAPK13* (#L-003591-00-0005), *MAP2K3* (#L-003509-00-0005), *MAP2K6* (#L-003967-00-0005), *FOS* (#L-003265-00-0005), *HSBP1* (#L-005269-00-0005 and #LQ-005269-00-0005) and siGENOME *MAP2K4* (#M-003574-02-0005). ON-TARGETplus Non-targeting Control Pool (#D-001810-10-20) was used as a control. All siRNAs were purchased from Dharmacon and were used at 20 nM. Transfection of siRNA was performed overnight using HiPerFect reagent (QIAGEN) according to the manufacturer’s reverse transfection protocol and maintained for a total of 48 h prior to serum starvation.

### Quantitative reverse transcription PCR

To test IL-6 transcription levels following c-Fos knockdown, TR146 cells (4 × 10^5^) in a 12-well tissue culture plate were treated as described above. 6 h following candidalysin treatment, mRNA was harvested using the QIAGEN RNeasy Kit (#74014, QIAGEN) and the RNase free DNase set (#79254, QIAGEN) following the manufacturer’s instructions. RNA isolated was measured using a NanoDrop 100 spectrophotometer. First-strand cDNA synthesis was performed using oligo(dt)15 primers (#C1101, Promega) and nuclease-free water under 70°C for 5 min. Reverse transcription was performed using the M-MLV Reverse Transcriptase kit (#M1701, Promega) in the presence of RNasin (#N2511, Promega) and deoxynucleotide triphosphates (dNTPs) (#U1205, #U1225, #U1215, #U1235, Promega) under 42°C for 60 min. qPCR was performed using the PowerUp SYBR Green Master Mix (#A25742, ThermoFisher Scientific) on a QuantStudio 7 No.2. The following primer sequences were from Sigma: *IL6* forward, 5'-AAAGAGGCACTGGCAGAAA-3'; *IL6* reverse, 5'-CAGGCAAGTCTCCTCATTGAA-3'; *FOS* forward, 5'-CTCCAGTGCCAACTTCATTC-3'; *FOS* reverse, 5'-ACTCCGAAAGGGTGAGG-3'; *GAPDH* forward, 5'-CTTCAACAGCGACACCCACT-3'; *GAPDH* reverse, 5'-GTGGTCCAGGGGTCTTACTC-3'; *IL6* and *FOS* expression were normalized to *GAPDH* and relative change to mRNA expression compared to non-targeting control was determined using the ΔΔCt method.

### Elisa

Following experimentation, cell culture supernatants were collected and centrifuged at 13000 rpm for 10 min. EGFR ligand concentration in cleared supernatants was determined using ELISA kits purchased from ELabscience (Epiregulin) and LSBio (Epigen) and performed following the manufacturer’s instructions. IL-6 concentration in cell extracts and supernatants of Fig. 2 was determined using a cell extraction buffer (#10334403, Fisher Scientific) and the ELISA kit purchased from Invitrogen (#KHC0061), while for fig. S7B the IL-6 ELISA kit was purchased from Abcam (#Ab178013) and performed according to the manufacturer’s instructions. The optical density of the plate was measured at 450 nm (TECAN, Magellan F50).

### Transcription factor DNA binding assay

TR146 cells were treated for 3 h prior to lysis to recover nuclear proteins using a nuclear protein extraction kit (Active Motif) according to the manufacturer’s instructions. Protein concentration was determined by BCA (Perbio Science) and 5 μg of nuclear extract was used to assess DNA binding activity of the c-Fos transcription factor using the TransAM transcription factor ELISA system (Active Motif) as previously described (7,8,20) and according to the manufacturer’s protocol. The optical density of the plate was measured at 450 nm (TECAN, Magellan F50).

### Murine oropharyngeal candidiasis model

Animal work was performed according to institutional and UK Home Office guidelines. This study was performed in accordance with the Project License and procedures were approved by King’s College London Animal Welfare and Ethical Review Body and the UK Home Office and the University of Pittsburgh IACUC. The animal care and use protocol adhered to the Animals (Scientific Procedures) Act 1986. *Il17ra^-/-^* mice (under MTA from Amgen), *Fos^fl/fl^* mice (MMRRC) and *K13^Cre^* mice (14) were on the C57BL/6 background and both sexes were infected at ages 6-10 weeks of age with the WT(CAF2-1) strain of *C. albicans.* Female, BALB/c mice aged 6-8 weeks were purchased from Charles River. Mice were co-housed for a minimum of 7 days prior to starting experiments. A previously described murine model of oropharyngeal candidiasis (61) was modified for investigating early infection events. Briefly, BALB/c mice were treated intra-peritoneally with 50 mg/kg of BIRB796 (in 5% DMSO + 40% PEG300 + 5% Tween 20 + 50% H2O) on day -1 pre-infection and for every 12 h up to 12 h pre-harvesting (a total of 4 doses for the 24 h mice and 6 doses for the 48 h mice). On day 0, mice were anaesthetized for ~75 min with an intra-peritoneal injection of 75 mg/kg ketamine and 1 mg/kg domitor, and a swab soaked in a 10^7^ cfu/ml *C. albicans* yeast (SC5314) culture in sterile saline was placed sub-lingually for 75 min. After 1 or 2 days, mice were sacrificed, the tongue excised and divided longitudinally in half. One half was weighed, homogenized by a Gentle MACS (Miltenyi Biotec) and plated on yeast extract peptone dextrose agar with ampicillin to derive quantitative *Candida* counts. The other half was processed for immunohistochemistry or RNA extraction. For the experiments where protein was used, whole tongues were homogenized, washed with cold PBS and resuspended in protein lysis buffer for 30 min on ice to be used for Western blotting as described above.

### Quantitative PCR for mouse samples

Tongue homogenates were prepared by a Gentle MACS Dissociator. Total mRNA was harvested using the QIAGEN RNeasy Plus Mini Kit (#74134, QIAGEN) following the manufacturer’s instructions. qRT-PCR was performed according to section above and by using the PowerUp SYBR Green Master Mix (#A25742, ThermoFisher Scientific) on a QuantStudio 7 No.2. For the p38 inhibition experiments the primer sequences used were from Sigma: *Il6* forward, 5'-GTTGCCTTCTTGGGACTGAT-3'; *Il6* reverse, 5'-TCTGCAAGTGCATCATCGT-3'; *Fos* forward, 5'-CAGCCTTTCCTACTACCATTCC-3'; *Fos* reverse, 5'-TGACACGGTCTTCACCATTC-3'; *Cxcl1* forward, 5' -GCTGGGATTCACCTCAAGAA -3'; *Cxcl1* reverse, 5'-TGGCTATGACTTCGGTTTGG-3'; *Cxcl2* forward, 5'-CCTGCCAAGGGTTGACTT-3'; *Cxcl2* reverse, 5'-CCTTGAGAGTGGCTATGACTTC-3'; *Defb3* forward, 5'-GTCTCCACCTGCAGCTTT-3'; *Defb3* reverse, 5'-AACTCCACAACTGCCAATCT-3'; *Il-17a* forward, 5'-GACTCTCCACCGCAATGAA-3'; *Il-17a* reverse, 5'-TTCCCTCCGCATTGACAC-3'; *Acta1* forward, 5'-CTGTGTTCCCATCCATCGT-3'; *Acta1* reverse, 5'-CACACGCAGCTCATTGTAGA-3'. *Il6* and *Fos* expression were normalized to *Acta1* and relative change to mRNA expression was determined using the ΔCt method. For the *Fos* knockout experiments total mRNA was subtracted by Trizol (Qiagen) followed by further purification using RNeasy kits (Qiagen). cDNA was generated using a SuperScript III First Strand Synthesis System (Invitrogen). Relative quantification of mRNA expression was determined by real-time PCR with SYBR green (Quanta BioSciences) normalized to *Gapdh* on the Applied Biosystems 7300 platform. Primers were purchased from QuantiTect (Qiagen).

### Immunohistochemistry

Mouse tongue halves were fixed for 24 h in 10% v/v neutral buffered formalin and processed to paraffin using a Tissue-Tek VIP® 6 AI. 3 μm tissue sections were cut, dried overnight at room temperature and baked at 60°C for 1 h prior to immunohistochemical staining for S100Α9 (2B10 clone) (#ab105472, abcam) on a Roche Ventana Discovery Ultra autostainer. Slides were counterstained with haematoxylin using a Tissue-Tek Prisma® automated slide stainer. Slides were imaged using the Zeiss Axio Scan.Z1 slide scanner and image analysis and quantification was performed using the QuPath 0.2.2 software (96). For the c-Fos staining, paraffin embedded sections were stained as described in the Cell Signaling immunofluorescence protocol (https://www.cellsignal.com/learn-and-support/protocols/protocol-ihc-paraffin). Images were obtained on an EVOS FL microscope (Life Technologies).

### Flow Cytometry for mouse bone marrow cells

Bone marrow was harvested from femurs and red blood cells were lysed (RBC lysis buffer, Biolegend). Bone marrow cells were blocked with TruStain FcX (Clone 93, Biolegend) and stained with anti-mouse CD45.2 (Clone 104, Biolegend) or IgG2a κ Isotype (Clone MOPC-173, Biolegend), anti-mouse CD11b (Clone M1/70, Biolegend) or IgG2b, κ Isotype (Clone RTK4530, Biolegend), anti-mouse Ly6G (Clone 1A8, Biolegend) or IgG2a, κ Isotype (Clone RTK2758, Biolegend) and anti-mouse Ly6C (Clone HK1.4, Biolegend) or IgG2c, κ Isotype (Clone RTK4174, Biolegend). Samples were analysed using a BD FACS Canto II and FlowJo software.

### Statistics

All graphs were created using GraphPad Prism®. Sample values were normalized to their respective control and data were analyzed by one sample t test compared to a hypothetical value = 1 (for comparisons between samples and their control) or by paired repeated measures one-way ANOVA with Tukey’s multiple comparisons test (for comparisons between samples). Where samples values were not normalized, these were compared to control values via paired t test. All in vivo data were analyzed via Mann-Whitney tests unless otherwise stated. In all cases, *P* < 0.05 was taken as significant. Fig. 8 was created with BioRender.

## Supplementary Material

Supplement Figures

## Data Availability

All data needed to evaluate the conclusions in the paper are present in the paper or the Supplementary Materials. Data and materials from the study will be made available under an MTA on request.
