# Supplementary material for: The Candida albicans toxin candidalysin mediates distinct epithelial inflammatory responses through p38 and EGFR-ERK pathways
Source: Sci Signal. Author manuscript; Available in PMC 2022 Apr 22. (PMC7612652; doi:10.1126/scisignal.abj6915)
Supplement: Supplement Figures [file EMS144405-supplement-Supplement_Figures.pdf]

Fig. S1. Candidalysin activates p38 independent of the EGFR-ERK1/2-c-Fos pathway. **A,**Graphical quantification of immunoblots as in Fig.1A. Graphs show means of three biological replicates + SD (for MKP1 data are from two biological replicates + range). **B,** Representative immunoblot showing phosphorylation levels of p38, ERK1/2 and Hsp27 30 min post-stimulation with increasing concentrations of candidalysin. Immunoblots are representative of three biological replicates. GAPDH is shown as a loading control. **C,** Graphical quantification of B. Data are expressed as percent change relative to cells treated with 70 μM of candidalysin. **D,** c-Fos binding at 3 h post-candidalysin (15 μM) stimulation in the presence of the EGFR inhibitors Gefitinib and PD153035 or the p38α, β inhibitor SB203580. Data are mean of two biological replicates + range (Gefitinib data is from one experiment) and are expressed as fold change relative to DMSO + candidalysin. **E,** Relative mRNA expression of *FOS* in TR146 cells treated with BIRB796 prior to candidalysin stimulation for 6 h. Data are mean of three biological replicates + SD. **F,** Representative immunoblot showing phosphorylation of p38 and ERK1/2 at indicated times post-candidalysin stimulation. Immunoblots are representative of two biological replicates. GAPDH is shown as a loading control. Statistical significance for A and E was quantified by one sample t test compared to a hypothetical value = 1. $^{*}P < 0.05$, $^{**}P < 0.01$, $^{***}P < 0.001$.

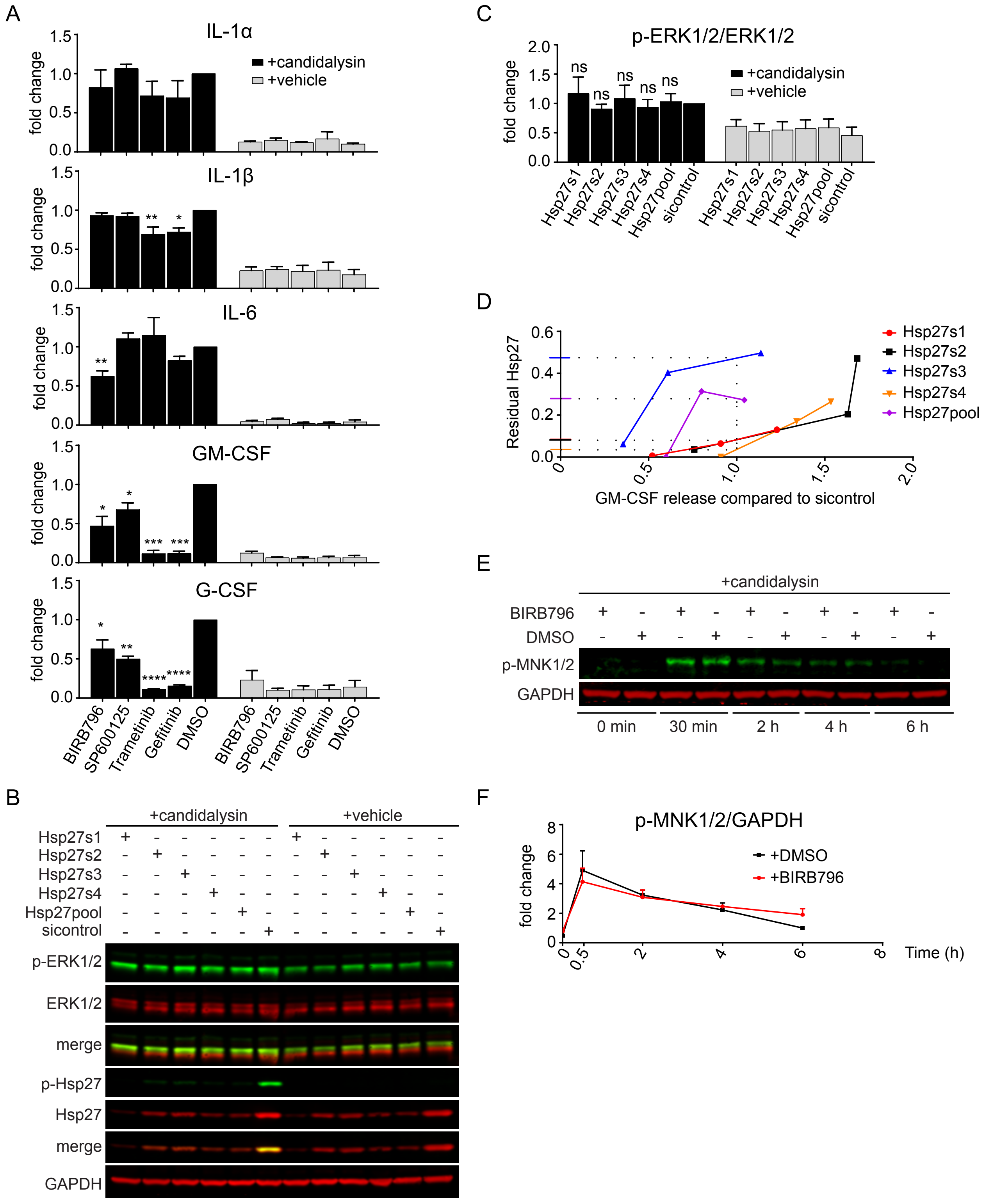
A
IL-1α
+candidalysin
+vehicle
fold change
IL-1β
IL-6
GM-CSF
G-CSF
BIRB796
SP600125
Trametinib
Gefitinib
DMSO
C
p-ERK1/2/ERK1/2
ns
Hsp27s1
Hsp27s2
Hsp27s3
Hsp27s4
Hsp27pool
sicontrol
D
Residual Hsp27
GM-CSF release compared to sicontrol
E
+candidalysin
BIRB796
DMSO
p-MNK1/2
GAPDH
0 min
30 min
2 h
4 h
6 h
B
p-ERK1/2
ERK1/2
merge
p-Hsp27
Hsp27
F
p-MNK1/2/GAPDH
+DMSO
+BIRB796
Time (h)

Fig. S2. p38 triggers cytokine release independent of ERK1/2. A, Release of proinflammatory cytokines and chemokines from TR146 cells 24 h post-candidalysin stimulation in the presence of the p38 inhibitors BIRB796 and SB203580, SP600125 (JNK inhibitor), Trametinib (MEK1/2 inhibitor of the ERK1/2 pathway) or Gefitinib (EGFR inhibitor). Graphs show means of three biological replicates + SD and are expressed as fold change relative to DMSO + candidalysin. **B,** Representative immunoblot showing total and phosphorylated levels of ERK1/2 and Hsp27 in TR146 cells transfected for 72 h with individual siRNAs and a pool siRNA for Hsp27 prior to candidalysin stimulation for 30 min. **C,** Graphical quantification of immunoblots as in B. Graphs show means of three biological replicates + SD and are expressed as fold change relative to siRNA control + candidalysin. **D,** Line graph showing residual Hsp27 protein expression as a consequence of Hsp27 knockdown versus GM-CSF release. Data show three individual biological repeats and are expressed at each point as fold change relative to siRNA control + candidalysin. **E,** Representative immunoblot showing phosphorylation of MNK1/2 at indicated times post-candidalysin stimulation and in the presence of BIRB796. Immunoblots for B and E are representative of three biological replicates. GAPDH is shown as a loading control. **F,** Graphical quantification of immunoblots as in E. Line graph shows the mean + SD of three biological replicates which are expressed as ratios of p-MNK1/2/GAPDH. Statistical significance in (A) and (C) was quantified by one sample t test compared to a hypothetical value = 1. $^{*}P < 0.05$, $^{**}P < 0.01$, $^{***}P < 0.001$, $^{****}P < 0.0001$.

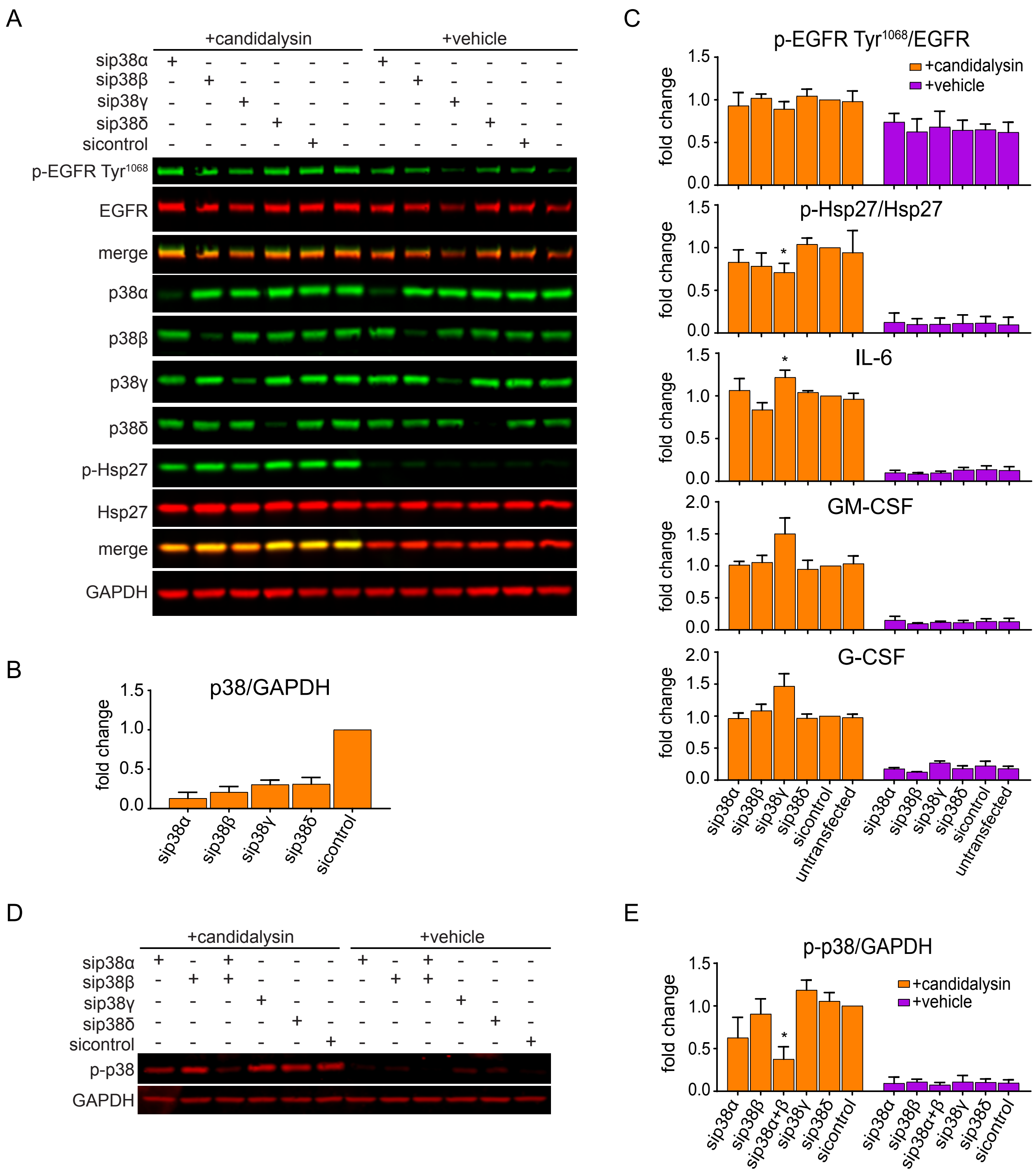
A
+candidalysin
+vehicle
sip38α
sip38β
sip38γ
sip38δ
sicontrol
p-EGFR Tyr1068
EGFR
merge
p38α
p38β
p38γ
p38δ
p-Hsp27
Hsp27
merge
GAPDH
B
p38/GAPDH
fold change
sip38α
sip38β
sip38γ
sip38δ
sicontrol
C
p-EGFR Tyr1068/EGFR
+candidalysin
+vehicle
p-Hsp27/Hsp27
IL-6
GM-CSF
G-CSF
fold change
untransfected
D
p-p38
GAPDH
E
p-p38/GAPDH
sip38α+β

Fig. S3. p38 presents functional redundancy among its isoforms. A, Representative immunoblot showing phosphorylation of EGFR on Tyr^{1068}, Hsp27 and total protein expression of p38α, β, γ and δ in TR146 cells transfected for 72 h with siRNAs for p38α, β, γ or δ prior to candidalysin stimulation for 30 min. **B,** Graphical quantification of knockdown efficiency (p38α=87.5%, p38β=80%, p38γ=70%, p38δ=70%) of p38 isoforms as in A. Columns represent total protein expression of each p38 isoform versus GAPDH following respective transfections. **C,** Graphical quantification of EGFR and Hsp27 phosphorylation as in A and release of proinflammatory cytokines and chemokines from cells transfected as in A prior to candidalysin stimulation for 24 h. **D,** Representative immunoblot showing phosphorylation of p38 in cells treated as in A. **E,** Graphical quantification of p-p38 versus GAPDH expression as in D. All immunoblots are representative of three biological replicates. GAPDH is shown as a loading control. Graphs in panels B, C and E show means of three biological replicates + SD and are expressed as fold change relative to siRNA control + candidalysin. Statistical significance was quantified by one sample t test compared to a hypothetical value = 1. $*P < 0.05$.

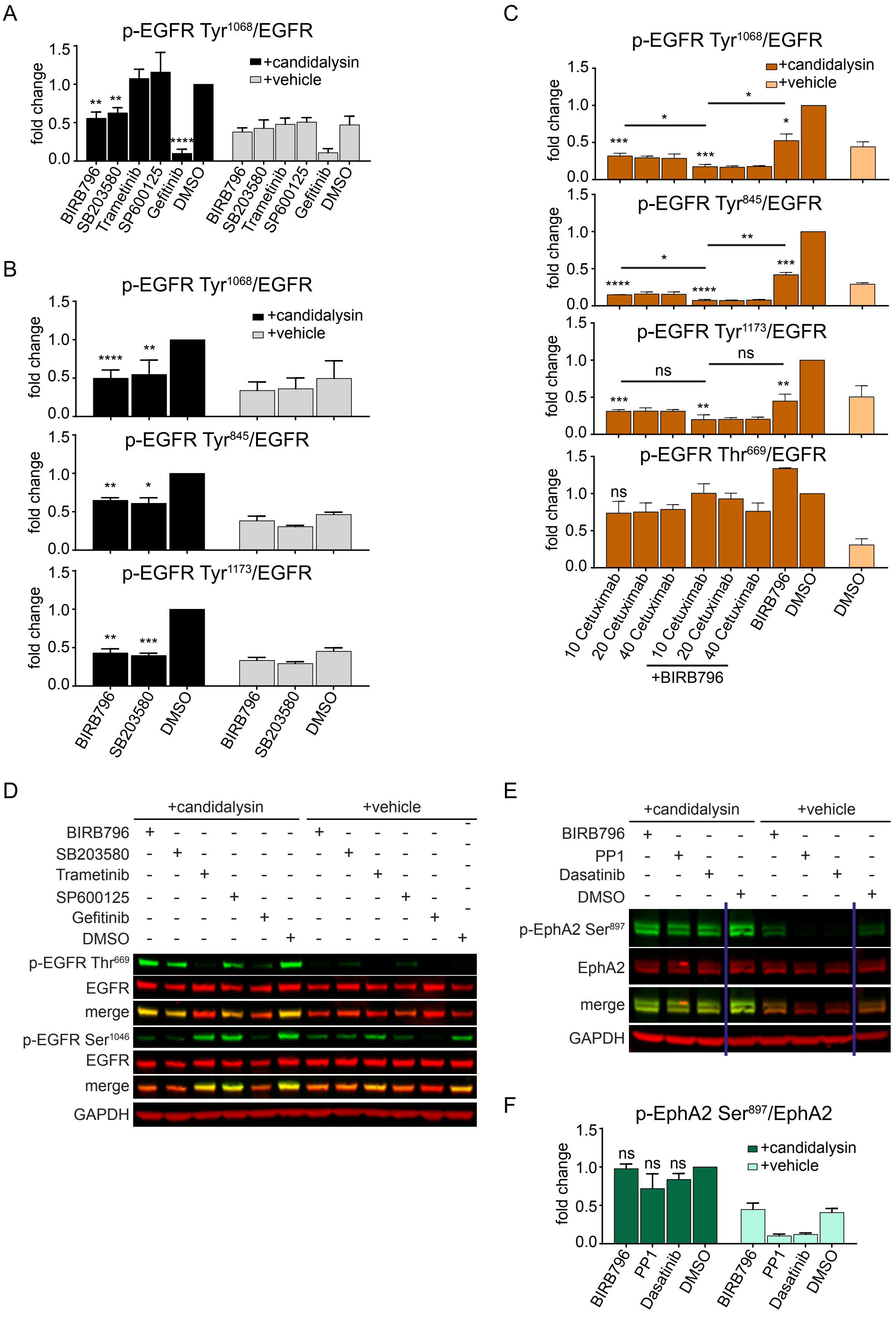
A
p-EGFR Tyr1068/EGFR
+candidalysin
+vehicle
fold change
1.5
1.0
0.5
0.0
**
**
****
BIRB796
SB203580
Trametinib
SP600125
Gefitinib
DMSO
B
p-EGFR Tyr1068/EGFR
+candidalysin
+vehicle
****
**
p-EGFR Tyr845/EGFR
**
*
p-EGFR Tyr1173/EGFR
**
***
BIRB796
SB203580
DMSO
C
p-EGFR Tyr1068/EGFR
+candidalysin
+vehicle
***
*
***
*
*
p-EGFR Tyr845/EGFR
****
*
****
**
***
p-EGFR Tyr1173/EGFR
***
ns
**
ns
**
p-EGFR Thr669/EGFR
ns
10 Cetuximab
20 Cetuximab
40 Cetuximab
10 Cetuximab
20 Cetuximab
40 Cetuximab
BIRB796
DMSO
DMSO
+BIRB796
D
+candidalysin
+vehicle
BIRB796
SB203580
Trametinib
SP600125
Gefitinib
DMSO
p-EGFR Thr669
EGFR
merge
p-EGFR Ser1046
EGFR
merge
GAPDH
E
+candidalysin
+vehicle
BIRB796
PP1
Dasatinib
DMSO
p-EphA2 Ser897
EphA2
merge
GAPDH
F
p-EphA2 Ser897/EphA2
+candidalysin
+vehicle
fold change
ns
ns
ns
BIRB796
PP1
Dasatinib
DMSO

Fig. S4. p38 contributes to candidalysin-induced EGFR phosphorylation via a ligand-independent manner. A, Graphical quantification of immunoblots as in Fig. 1A. **B,** Graphical quantification of immunoblots as in Fig. 3A. **C,** Graphical quantification of immunoblots as in Fig. 3B. **D,** Representative immunoblot showing phosphorylation of EGFR on Thr^{669} and Ser^{1046} in TR146 cells treated for 30 min with candidalysin in the presence of BIRB796, SB203580 (p38 inhibitors), Trametinib (MEK1/2 inhibitor of the ERK1/2 pathway), SP600125 (JNK inhibitor) or Gefitinib (EGFR inhibitor). Immunoblots are representative of two biological replicates. **E,** Representative immunoblot showing phosphorylation of EphA2 on Ser^{897} in TR146 cells treated for 30 min with candidalysin in the presence of BIRB796, PP1 (SFK inhibitor) or Dasatinib (Src-selective inhibitor). Immunoblots are representative of three biological replicates. GAPDH is shown as a loading control. Lines represent intervening gel lanes which are deleted. **F,** Graphical quantification of immunoblots as in E. Graphs in panels A, B, C and F show means of three biological replicates + SD and are expressed as fold change relative to DMSO + candidalysin. Statistical significance in A, B and F was quantified by one sample t test compared to a hypothetical value = 1. Statistical significance in C was quantified by one sample t test compared to a hypothetical value = 1 and by paired repeated measures one-way ANOVA with Tukey's multiple comparisons test as indicated in the graphs. *P < 0.05, **P < 0.01, ***P < 0.001, ****P < 0.0001.

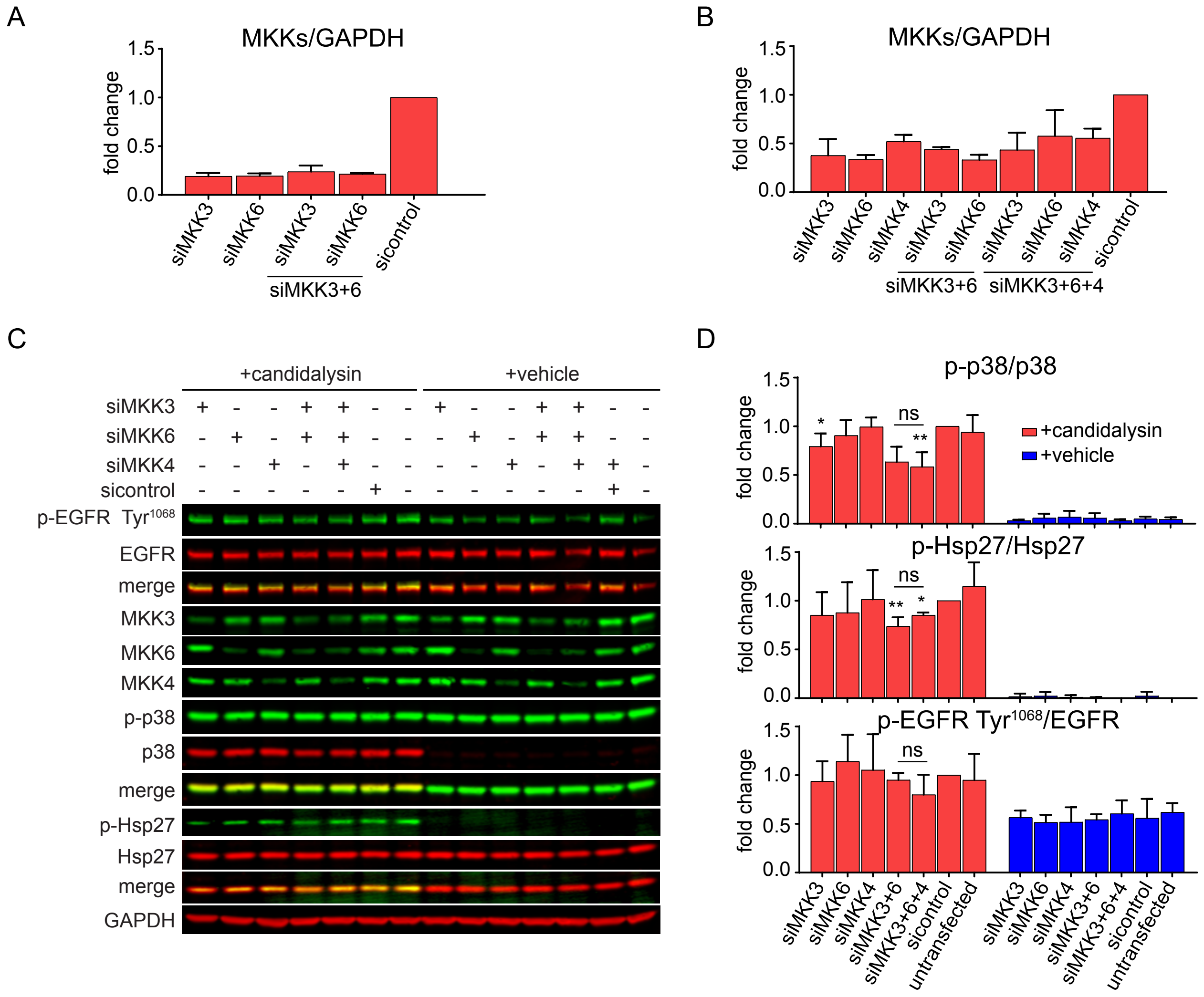

Fig. S5. MKK4 does not have a profound role in the candidalysin-induced p38 activation. **A,** Graphical quantification of immunoblots as in Fig. 4A showing knockdown efficiency (MKK3=81%, MKK6=81%). Columns represent total protein expression of each MKK versus GAPDH following single and double (siMKK3+6) transfections. **B,** Graphical quantification of immunoblots as in C showing knockdown efficiency (MKK3=63%, MKK6=67%, MKK4=48%). Columns represent total protein expression of each MKK versus GAPDH post single, double (siMKK3+6) and triple (siMKK3+6+4) transfections. **C,** Representative immunoblot showing phosphorylation of EGFR on Tyr^{1068}, p38 and Hsp27 and total protein expression of MKK3, 6 and 4 in TR146 cells transfected for 72 h with siRNAs for MKK3, MKK6 and MKK4 prior to candidalysin stimulation for 30 min. GAPDH is shown as a loading control. Data are representative of three biological replicates. **D,** Graphical quantification of immunoblots as in C. Graphs show means of three biological replicates + SD and are expressed as fold change relative to siRNA control + candidalysin. Statistical significance in D was quantified by one sample t test compared to a hypothetical value = 1 and by paired repeated measures one-way ANOVA with Tukey's multiple comparisons test as indicated in the graphs. $^{*}P < 0.05$, $^{**}P < 0.01$.

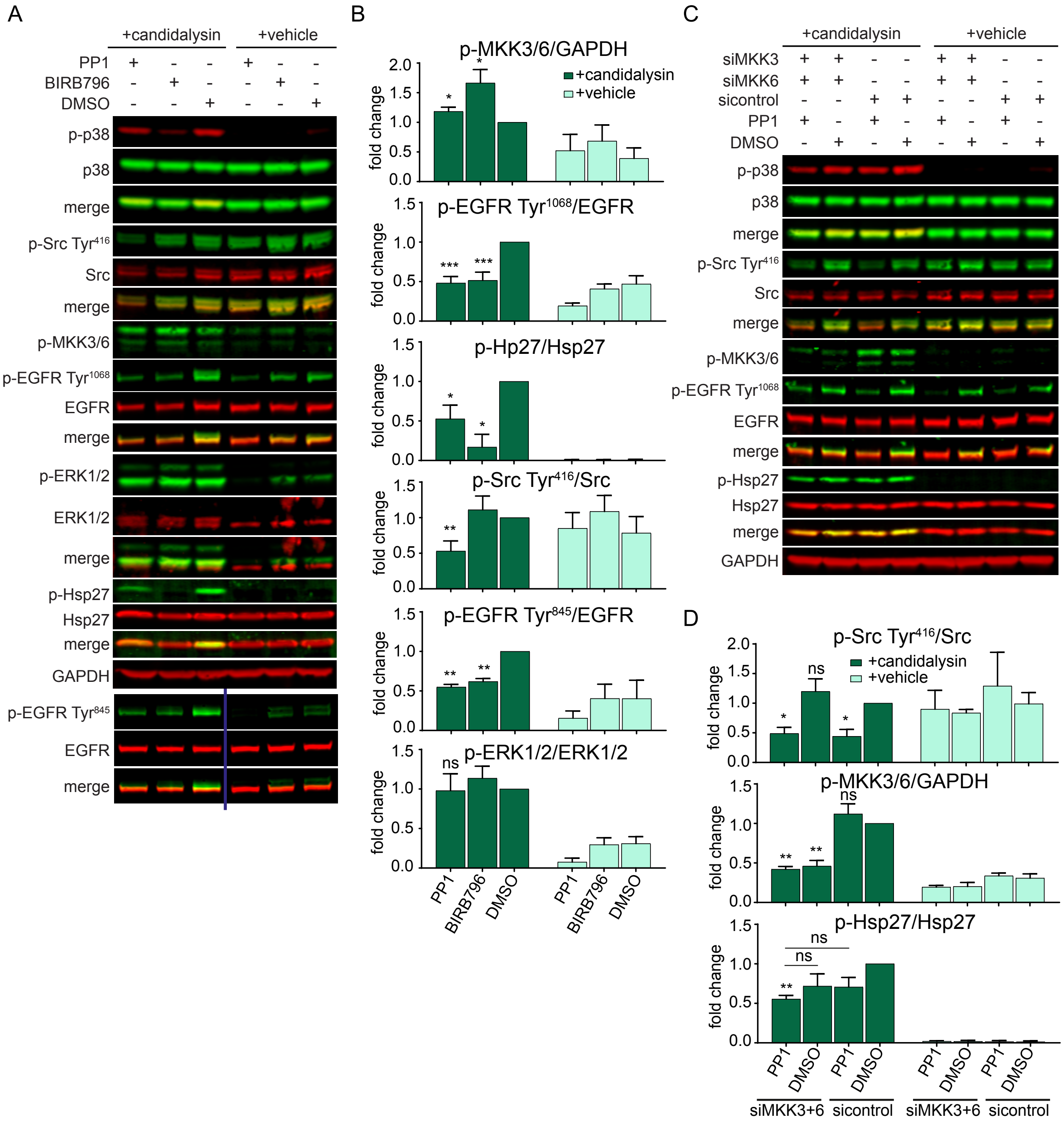
A
+candidalysin
+vehicle
PP1
BIRB796
DMSO
p-p38
p38
merge
p-Src Tyr416
Src
merge
p-MKK3/6
p-EGFR Tyr1068
EGFR
merge
p-ERK1/2
ERK1/2
merge
p-Hsp27
Hsp27
merge
GAPDH
p-EGFR Tyr845
EGFR
merge
B
p-MKK3/6/GAPDH
+candidalysin
+vehicle
fold change
p-EGFR Tyr1068/EGFR
p-Hp27/Hsp27
p-Src Tyr416/Src
p-EGFR Tyr845/EGFR
p-ERK1/2/ERK1/2
ns
PP1
BIRB796
DMSO
C
+candidalysin
+vehicle
siMKK3
siMKK6
sicontrol
PP1
DMSO
p-p38
p38
merge
p-Src Tyr416
Src
merge
p-MKK3/6
p-EGFR Tyr1068
EGFR
merge
p-Hsp27
Hsp27
merge
GAPDH
D
p-Src Tyr416/Src
+candidalysin
+vehicle
fold change
ns
p-MKK3/6/GAPDH
p-Hsp27/Hsp27
PP1
DMSO
siMKK3+6
sicontrol

Fig. S6. Src-family kinases contribute to candidalysin-induced p38 activation independently of MKKs. A, Representative immunoblot showing phosphorylation of p38, Src on Tyr^{416}, MKK3/6, EGFR on Tyr^{1068} and Tyr^{845}, ERK1/2 and Hsp27 30 min post-candidalysin stimulation in the presence of PP1 (Src-family inhibitor) or BIRB796 (pan-p38 inhibitor). **B,** Graphical quantification of immunoblots as in A. **C,** Representative immunoblot showing phosphorylation of p38, Src on Tyr^{416}, MKK3/6, EGFR on Tyr^{1068} and Hsp27 in TR146 cells transfected for 72 h with siRNA for MKK3 and MKK6 and/or treated with PP1 prior to candidalysin stimulation for 30 min. All immunoblots are representative of three biological replicates. GAPDH is shown as a loading control. Lines represent intervening gel lanes which are deleted. **D,** Graphical quantification of immunoblots as in C. Graphs show means of three biological replicates + SD and are expressed as fold change relative to DMSO + candidalysin for B or DMSO + siRNA control + candidalysin for D. Statistical significance in B was quantified by one sample t test compared to a hypothetical value = 1. Statistical significance in D was quantified by one sample t test compared to a hypothetical value = 1 and by paired repeated measures one-way ANOVA with Tukey's multiple comparisons test as indicated in the graphs. *$P < 0.05$, **$P < 0.01$, ***$P < 0.001$.

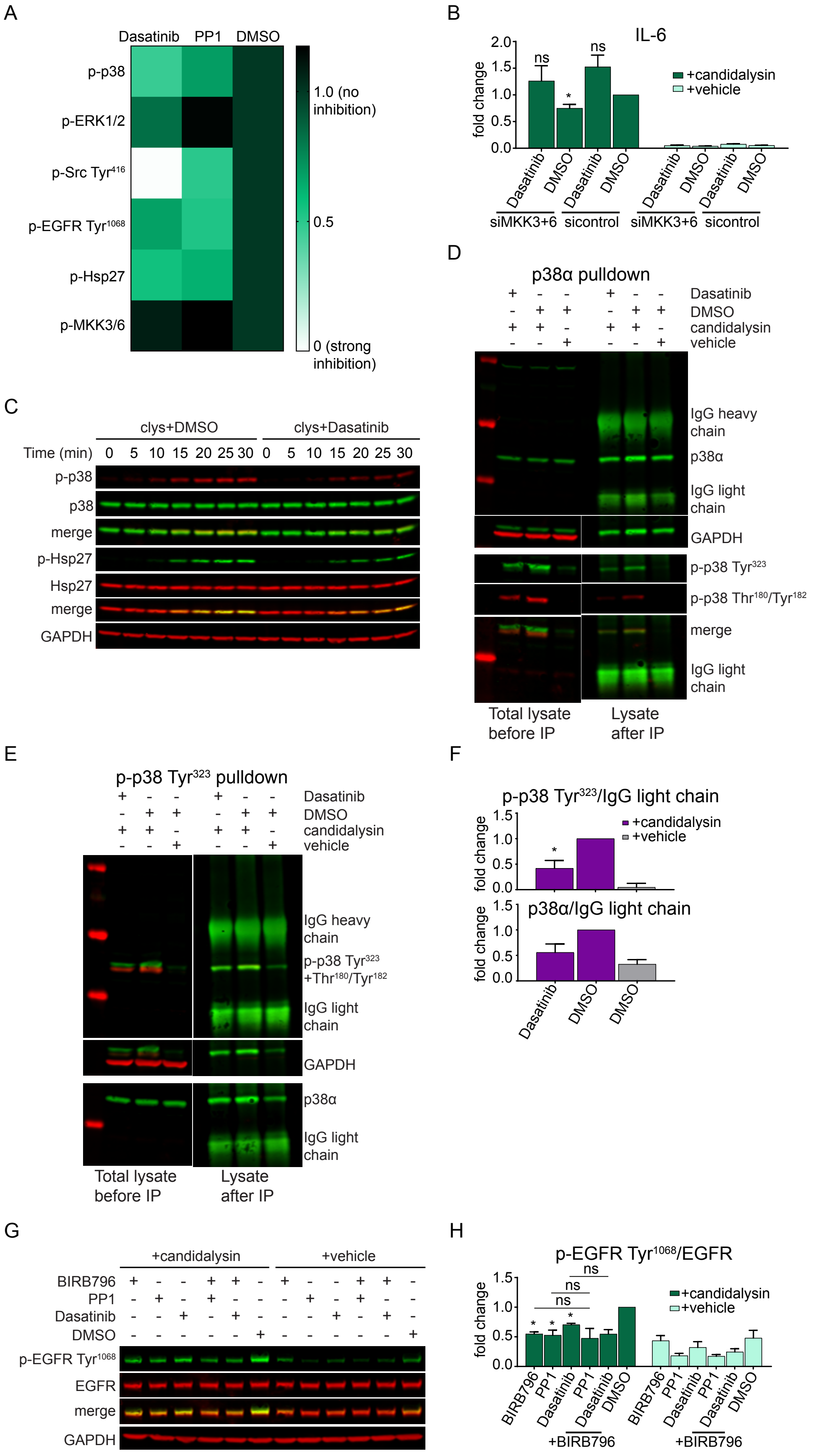
A
Dasatinib PP1 DMSO
p-p38
p-ERK1/2
p-Src Tyr416
p-EGFR Tyr1068
p-Hsp27
p-MKK3/6
1.0 (no inhibition)
0.5
0 (strong inhibition)
B
IL-6
fold change
+candidalysin
+vehicle
siMKK3+6 sicontrol siMKK3+6 sicontrol
C
clys+DMSO clys+Dasatinib
Time (min) 0 5 10 15 20 25 30 0 5 10 15 20 25 30
p-p38
p38
merge
p-Hsp27
Hsp27
merge
GAPDH
D
p38α pulldown
Dasatinib
DMSO
candidalysin
vehicle
IgG heavy chain
p38α
IgG light chain
GAPDH
p-p38 Tyr323
p-p38 Thr180/Tyr182
merge
IgG light chain
Total lysate before IP
Lysate after IP
E
p-p38 Tyr323 pulldown
Dasatinib
DMSO
candidalysin
vehicle
IgG heavy chain
p-p38 Tyr323 +Thr180/Tyr182
IgG light chain
GAPDH
p38α
IgG light chain
Total lysate before IP
Lysate after IP
F
p-p38 Tyr323/IgG light chain
+candidalysin
+vehicle
p38α/IgG light chain
fold change
Dasatinib DMSO DMSO
G
+candidalysin +vehicle
BIRB796
PP1
Dasatinib
DMSO
p-EGFR Tyr1068
EGFR
merge
GAPDH
H
p-EGFR Tyr1068/EGFR
fold change
+candidalysin
+vehicle
BIRB796 PP1 Dasatinib PP1 Dasatinib DMSO
+BIRB796

Fig. S7. Src induces phosphorylation of p38 on Tyr^{323} following candidalysin stimulation. **A,** Heatmap representation showing phosphorylation of p38, ERK1/2, Src on Tyr^{416}, EGFR on Tyr^{1068}, Hsp27 and MKK3/6 30 min post-candidalysin stimulation in the presence of Dasatinib (Src-selective inhibitor) or PP1 (Src-family inhibitor). **B,** Release of IL-6 from TR146 cells transfected for 72 h with siRNA for MKK3 and MKK6 and/or treated with Dasatinib prior to candidalysin stimulation for 24 h. Graph shows means of three biological replicates + SD and is expressed as fold change relative to DMSO + siRNA control + candidalysin. **C,** Representative immunoblot showing phosphorylation of p38 and Hsp27 in TR146 cells treated with Dasatinib or DMSO and harvested at the indicated times post-candidalysin stimulation. **D,** (Right) Representative immunoblots demonstrating amounts of p38α and p-p38 on Tyr^{323} and Thr^{180}/Tyr^{182} after immunoprecipitation of p38α in lysates of TR146 cells stimulated with candidalysin for 30 min and in the presence of Dasatinib. (Left) Immunoblots of lysates prior to immunoprecipitation, demonstrating equal amounts of input protein. Immunoblots are representative of three biological replicates. GAPDH is shown as a loading control of the total lysates prior to immunoprecipitation. **E,** (Right) Representative immunoblots demonstrating amounts of p38α and p-p38 on Tyr^{323} and Thr^{180}/Tyr^{182} after immunoprecipitation of p-p38 Tyr^{323} in lysates of TR146 cells stimulated with candidalysin for 30 min and in the presence of Dasatinib. (Left) Immunoblots of lysates prior to immunoprecipitation, demonstrating equal amounts of input protein. Immunoblots are representative of two biological replicates. GAPDH is shown as a loading control of the total lysates prior to immunoprecipitation. **F,** Graphical quantification of immunoblots as in D-E. Data are normalized to the IgG light chain and are means of three + SD and two biological replicates + range respectively. Graphs in panels B and D are expressed as fold change relative to DMSO + candidalysin. Statistical significance was quantified by one sample t test compared to a hypothetical value = 1. **G,** Representative immunoblot showing phosphorylation of EGFR on Tyr^{1068} in TR146 cells treated with different combinations of BIRB796 (pan-p38 inhibitor), PP1 and Dasatinib prior to candidalysin stimulation for 30 min. **H,** Graphical quantification of immunoblots as in G. Graph shows means of three biological replicates + SD and is expressed as fold change relative to DMSO + candidalysin. Statistical significance in B and H was quantified by one sample t test compared to a hypothetical value = 1 and by paired repeated measures one-way ANOVA with Tukey's multiple comparisons test as indicated in the graphs. $^*P < 0.5$.

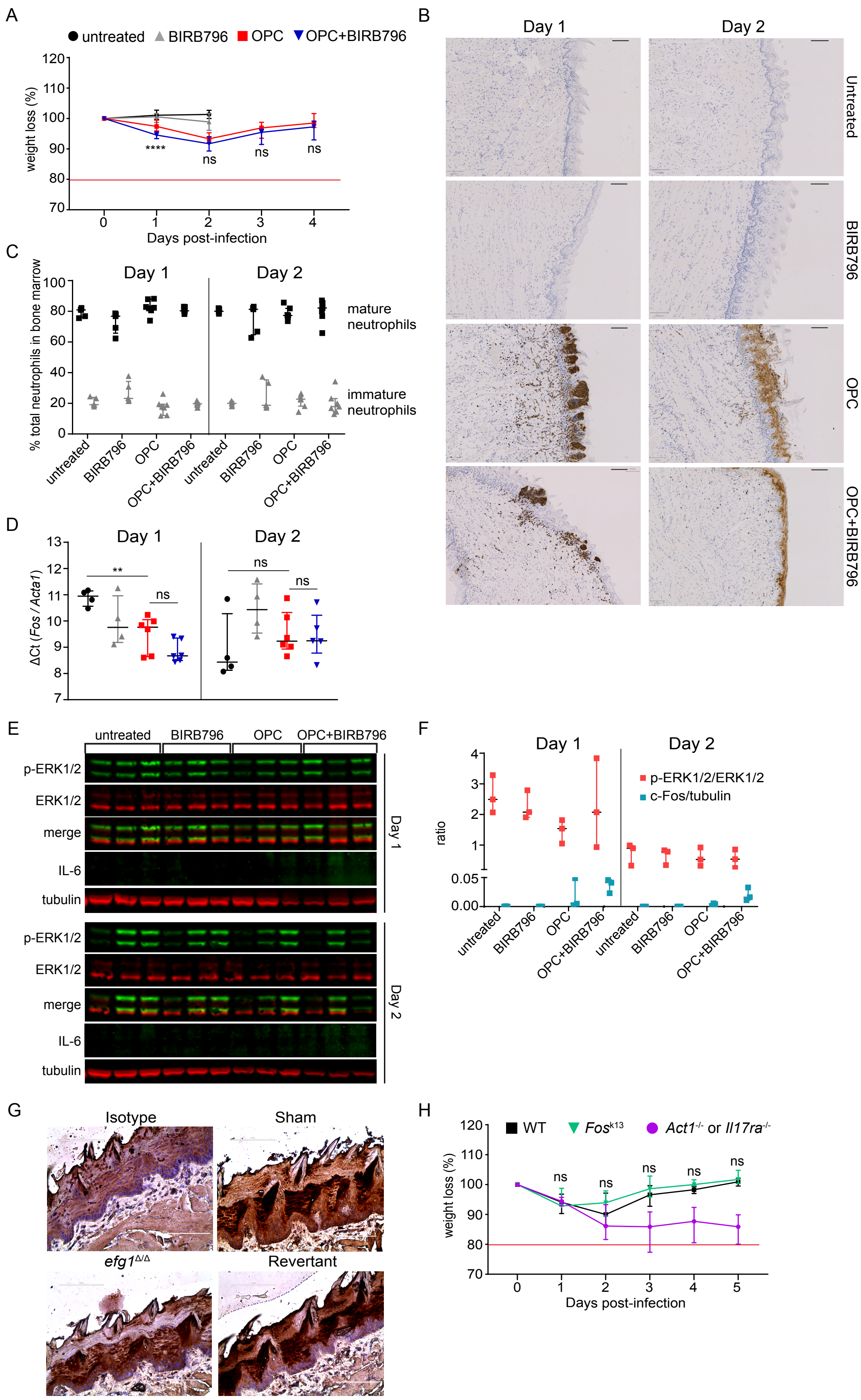
A
untreated BIRB796 OPC OPC+BIRB796
weight loss (%)
Days post-infection
**** ns ns ns
B
Day 1
Day 2
Untreated
BIRB796
OPC
OPC+BIRB796
C
Day 1
Day 2
% total neutrophils in bone marrow
mature neutrophils
immature neutrophils
untreated BIRB796 OPC OPC+BIRB796
D
Day 1
Day 2
ΔCt (Fos / Acta1)
** ns ns ns
E
untreated BIRB796 OPC OPC+BIRB796
p-ERK1/2
ERK1/2
merge
IL-6
tubulin
Day 1
Day 2
F
Day 1
Day 2
p-ERK1/2/ERK1/2
c-Fos/tubulin
ratio
G
Isotype
Sham
efg1Δ/Δ
Revertant
H
WT Fosk13 Act1-/- or Il17ra-/-
weight loss (%)
Days post-infection
ns ns ns ns ns

Fig. S8. p38 is required for early clearance of *C. albicans* during OPC independent of c-Fos. Wild-type (Balb/c) immunocompetent mice were treated with either BIRB796 (pan-p38 inhibitor) or vehicle control, and orally inoculated with *C. albicans*. **A,** Mouse weights were monitored daily and percentage loss relative to day 0 is shown for each time point. Results are from 11-30 mice per group obtained over two independent experiments (one for day 4) (multiple Mann-Whitney tests). **B,** Immunohistochemistry (IHC) in tongues of untreated, BIRB796 treated, and *C. albicans* infected mice with or without BIRB796, showing neutrophil recruitment 1 and 2 days post infection (p.i.). The tongues were stained with anti-S100A9 antibody (2B10) and hematoxylin. Scale bars at 100 μm. Results are representative of 4-6 mice per group obtained over two independent experiments. **C,** Percentage of mature (CD45.2^{+} CD11b^{+} Ly6C^{INT} Ly6G^{HI}) and immature (CD45.2^{+} CD11b^{+} Ly6C^{INT} Ly6G^{LOW}) neutrophils in bone marrow isolated from mice femurs. Results are median ± interquartile range of 5-7 mice per group obtained over two independent experiments (two-way ANOVA). **D,** Relative mRNA expression of *Fos* in tongue homogenates 1 and 2 days p.i. mRNA levels were determined by ΔCT method and normalized to *Acta1*. Results are median ± interquartile range of 4-6 mice per group obtained over two independent experiments (Mann-Whitney tests). **E,** Immunoblot from a single cohort of mice showing phosphorylation of ERK1/2 and IL-6 expression from tongues of mice orally inoculated with *C. albicans* with or without BIRB796 and their respective controls 1 (top) and 2 (bottom) days p.i. Tubulin is shown as a loading control. **F,** Densitometric analysis of ERK1/2 phosphorylation and c-Fos induction as shown in G and Fig. 6H respectively. Data are expressed as ratios of p-ERK1/2/ERK1/2 and c-Fos/tubulin. Results are median ± interquartile range of 3 mice per group from a single cohort of mice. **G,** Wild-type (C57BL/6) mice were orally challenged by *efg1*Δ/Δ strain of *C. albicans*. The mice were harvested at 24 h p.i. for IHC staining. c-Fos expression was analyzed by IHC staining on paraffin fixed sections from tongues. Images are representative of at least two sections from individual mice. Scale bars at 100 μm. **H,** *Fos*^{k13} mice were sublingually infected with *C. albicans* for 5 days. Mouse weights were monitored daily and percentage loss relative to day 0 is shown for each time point. Results are from 7-9 mice per group obtained over two independent experiments (multiple Mann-Whitney tests). Each symbol represents one mouse. $**P < 0.01$, $****\ P < 0.0001$.
